# Cross-crop generalization in multi-species leaf disease recognition

**DOI:** 10.3389/fpls.2026.1970636

**Published:** 2026-10-29

**Authors:** Zhaopeng Cai, Nadia Farhana, Jiawei Li, Asif Mahbub Karim, Lizheng Guo, Junhui Cao

**Affiliations:** 1 School of Computer and Artificial Intelligence, Research Center of Smart City and Big Data Engineering of Henan Province, Research Center of Intelligent Campus Application Engineering of Henan Province, Henan University of Urban Construction, Pingdingshan, China; 2 Binary Graduate School, Binary University of Management & Entrepreneurship, Puchong, Selangor, Malaysia; 3 Department of Business Administration, Stamford University Bangladesh, Dhaka, Bangladesh; 4 School of Computer and Artificial Intelligence, Henan University of Urban Construction, Pingdingshan, China

**Keywords:** cross-crop generalization, disentangled representation learning, functional-domain separation, phytopathological signatures, plant disease recognition

## Abstract

**Introduction:**

Plant diseases are major biotic stress factors whose visual symptoms may be conserved across taxonomic families, though the extent remains unquantified. Cross-crop leaf disease recognition requires architectures that generalize to species absent from training, but existing methods lack a theoretically grounded criterion for ensuring cross-species transferability.

**Methods:**

This study proposes functional-domain separation as such a criterion, derived from the disentangled representation learning hypothesis, and implements it as the leaf-disease composite feature interaction network (LCFIN), a configurable multi-branch feature enhancement framework. LCFIN assigns a two-level Squeeze-and-Excitation mechanism to a channel calibration domain and an MLP-Mixer head to a spatial interaction domain on an EfficientNet-B3 backbone, with dual-weighted sampling addressing cross-level class imbalance. It is trained for main fruit and vegetable leaf disease identification (MFVLDI) on three families (Rosaceae, Vitaceae, Solanaceae) and evaluated under a frozen-representation linear-probe protocol on two unseen crops, maize of Poaceae and potato of Solanaceae.

**Results:**

LCFIN reaches 88.99% accuracy on maize and 95.23% on potato under matched acquisition conditions. Across five independent group-aware partitions the in-domain accuracy is 99.93% ± 0.05%, with 19 misclassified test images among 27,937. A duplicate and near-duplicate audit bounds the accuracy inflation attributable to data redundancy at a few hundredths of a percentage point. Ablation across the five partitions shows the two domains to be functionally complementary rather than redundant, but the interaction is sub-additive, with a residual of 0.04 percentage points below the additive prediction.

**Discussion:**

These findings are consistent with the learned features capturing phytopathological regularities, although this observation remains descriptive rather than causal. They support the hypothesis that functional-domain separation induces implicit feature disentanglement and enables capture of phytopathological signatures shared across the tested families, without demonstrating that they transcend taxonomic boundaries. Because the extractor is frozen and only a linear head is fitted, extending the model to a further crop costs far less than training a network from scratch.

## 1 Introduction

Despite significant progress in single-crop disease recognition, the prevailing approach of training species-specific recognition systems faces a fundamental scalability barrier. With over 100 economically important crop species cultivated worldwide and thousands of associated plant diseases documented in phytopathological literature, deploying individual recognition architectures for each crop-disease combination is impractical in terms of data collection, computational overhead, and system maintenance. This limitation becomes particularly acute in smallholder agricultural systems across diverse agroecological zones, where multiple crop species are cultivated simultaneously and access to specialized diagnostic infrastructure is limited. A cross-crop generalization approach, wherein a single architecture can recognize diseases across taxonomically diverse species without species-specific retraining, would address this scalability challenge directly. However, achieving such generalization requires moving beyond purely data-driven feature extraction toward architectures whose design principles align with the biological regularities underlying cross-species disease manifestation.

Leaf diseases constitute a major biotic stress factor affecting global crop production, causing significant yield losses and reducing produce quality across diverse agricultural systems. The ability to identify and manage these diseases across different crop species is fundamental to sustainable agriculture and food security. Traditional disease identification relies on visual inspection by trained agriculturalists, a process constrained by subjectivity, limited scalability, and delayed intervention. The rapid advancement of deep learning and computer vision has enabled automated, image-based disease identification, attracting growing research attention in the field of plant pathology and precision agriculture. From a phytopathological perspective, plant pathogens across plant species trigger conserved programmed cell death pathways (Glazebrook, 2005; Jones and Dangl, 2006), producing visually similar lesion patterns such as necrotic spots and chlorotic haloes that are conserved across taxonomic families. This conservation raises a fundamental scientific question: whether deep learning architectures can be designed to capture these shared pathological regularities and generalize across plant families. From the perspective of plant-biotic interactions, fungal pathogens engage in co-evolutionary arms races with their hosts, yet the conserved defense pathways across plant families produce visually analogous symptoms that are conserved across taxonomic families (Glazebrook, 2005; Jones and Dangl, 2006). While molecular biology has established these conserved defense mechanisms at the cellular level, computational approaches offer a complementary perspective by testing whether cross-family conservation is detectable in visual symptom patterns across taxonomically diverse species. This study bridges the gap between molecular evidence and computational validation by testing whether architectures with appropriate design principles can learn transferable phytopathological signatures across plant families spanning Solanaceae, Vitaceae, Rosaceae, and Poaceae.

A central question in automated plant disease recognition is whether visual pathological features exhibit cross-species conservation, such that a recognition architecture trained on diseases of certain crop species can generalize to diseases of entirely unseen crop species without fine-tuning of the feature extractor. This question has significant implications for plant disease management: if cross-species generalization is achievable, it could reduce the substantial annotation and training costs associated with deploying recognition systems for new crops. From a representation learning perspective, this question is equally fundamental: whether architectural design principles can be formulated to disentangle disease-specific features from crop-specific morphological features, thereby enabling cross-species transfer (Bengio et al., 2013). This study investigates this question using three globally cultivated crop species: tomato, grape, and apple, selected for their high economic value, extensive planting coverage, and prevalent leaf diseases. The dataset comprises 12 categories of leaf diseases across these three crops.

Deep learning approaches using convolutional neural networks to automatically extract leaf lesion, texture, and color features have become a mainstream technical solution for intelligent disease prevention and control. However, most existing methods are optimized for single-crop scenarios and lack design criteria for ensuring cross-species transferability.

### 1.1 Single-crop recognition and lightweight network design

Existing lightweight research is mostly based on native lightweight networks such as ShuffleNet and MobileNeXt, or pruning and distillation transformations for general networks such as ResNet, reducing parameters through deep separable convolution, asymmetric convolution, or model pruning to adapt to mobile computing resource constraints.

Ji et al. (2025) optimized the depth-separable convolution structure of ShuffleNet V2 and introduced a coordinate attention mechanism to propose the CA-ShuffleNet V2 model. The model only contains 2.5M parameters with a computational overhead of 172.4 MFLOPs. The accuracy of the architecture is 2.8% higher than that of the original model in 16 types of disease identification in the PlantVillage dataset. The overall performance is better than AlexNet and VGG19, which can meet the real-time detection requirements of mobile terminals. Zhang (2025) improved ResNet for maize diseases to obtain AC-SK-ResNet. By optimizing residual structure, introducing SK attention, and asymmetric convolution, irregular disease spot features were extracted finely. Compared with the ResNet18 model, the volume compression was 77.01%, and the identification accuracy was 98.7%. Zou (2025) combined the advantages of convolution and Transformer to design ECVNet and then obtained lightweight LMNet through multipath pruning. The new model parameters and calculations are only 5.69% and 3.80% of the original model, and the accuracy on tomato datasets has a slight increase. Liu (2023) embedded CA attention, the Inception module, and hybrid convolution based on MobileNeXt to improve apple disease identification under complex field backgrounds. After improvement, the parameters of the model increase very little, and the identification accuracy increases by more than 1%. The PlaNet proposed by Khanna et al. (2024) does not require artificial design features and is trained using multi-scale leaf images. The average accuracy rate is 97.95% in three standard datasets and 18 mainstream network comparison experiments. The lightweight and versatility are good.

Most existing lightweight models are optimized for single crops and lack a general backbone network for multiple crops such as apples, grapes, and tomatoes. EfficientNet balances the depth, width, and input resolution of the network through a compound scaling strategy and shows better generalization on multi-scale and multi-category visual tasks. However, current lightweight network research has not proposed architectural design principles that guide cross-species generalization; model improvements remain confined to optimizing performance within single-crop scenarios. Therefore, EfficientNet-B3 is selected as the backbone network in this study.

### 1.2 Attention mechanism design for disease feature enhancement

When identifying leaf diseases, we often encounter different sizes of leaf disease spots, high similarity between disease texture and leaf body, and background interference such as weeds and soil in field shooting. Therefore, most of the existing research introduces channel, spatial, or mixed attention into convolutional networks and highlights the disease area and suppresses the background noise by feature weighting.

Marriam et al. (2024) took coffee leaf disease images as research objects. Based on the ResNet50 fusion space-channel attention mechanism and the CenterNet detection framework, the CoffeeNet model was constructed. The network screened key pixels of coffee leaf lesions using attention. The classification accuracy rate reached 98.54%, and the lesion location mAP reached 0.97 on the complex field real coffee dataset. Ray et al. (2025) designed a DH-CNN double-head convolutional network, shared a feature layer to extract common leaf features uniformly, and matched with double classification heads to complete two tasks of crop species variety discrimination and disease identification at the same time. The attention mechanism runs through the whole process of feature extraction. On 13,576 unseen leaf samples, the leaf classification accuracy reaches 99.71%, and the disease identification accuracy reaches 99.26%, which verifies the practical value of the attention mechanism for multi-task feature screening. Tong (2023) proposed the ResNet34-CBAM network by embedding CBAM attention into the ResNet34 residual block. It improves the feature weight of tomato small disease spots by attention weight, suppresses irrelevant interference from leaf veins and other background features, and has better accuracy than the original network on ten tomato disease datasets. The Web-side identification system based on this model takes only 3.6s to identify a single picture. Chen (2023) proposed the CACPNET model, on the one hand, by optimizing the channel attention pool combination method to improve the ability to extract fine lesions, on the other hand, using L1 weight sorting to complete channel pruning, after pruning on PlantVillage and self-built peanut double datasets, the model parameters and calculations are reduced by more than 50% and the identification accuracy can reach 99.7%. Bi (2024) starts from the existing challenges of complex, noisy real-scene images, combines attention and a multi-scale feature fusion strategy based on ResNet50, and adopts a double-branch structure to splice deep and shallow disease features, effectively improving the identification reliability of dust and shadow interference samples in the field. Liang (2023) used the improved YOLOv5s for leaf target extraction based on a split task strategy, matched the DM-2 classification model to complete tomato disease detection, embedded an attention module into the feature pyramid layer, filtered non-leaf miscellaneous background, and its leaf detection accuracy reached 87.5% in a complex environment, and disease classification accuracy reached 98.20%.

However, most of the existing improved schemes are only suitable for disease feature extraction of a single crop and lack a universal attention design for differential leaf morphology. A fundamental limitation of existing attention mechanisms is the failure to distinguish functional domains: channel calibration and spatial interaction are mixed within the same module, causing gradient signals from different functional domains to interfere with one another. The Squeeze-and-Excitation (SE) module achieves adaptive calibration of channel weights through a compression and excitation mechanism, which does not depend on the morphological prior of specific crops and can adapt to disease feature screening in multi-crop scenarios, but addresses only the channel calibration domain without explicit spatial interaction modeling.

### 1.3 Cross-crop disease recognition and functional-domain separation

With the development of large-scale precision agriculture, a variety of crop species are often mixed in the same park, and the demand for general algorithms capable of identifying diseases across categories continues to increase, but the relevant research is still immature at this stage.

Bouacida et al. (2025) explored a common algorithm across crop species, abandoning the traditional idea of relying on the appearance of the whole leaf, using a lightweight Inception architecture to focus on the pixel features of local disease spots and remove the appearance differences caused by leaf varieties. The accuracy rate of this method reached 94.04% on PlantVillage multi-crop datasets, and the migration accuracy rate reached 97.13% on new crop datasets containing only simple disease categories that were not involved in training. It is proven that it is feasible to achieve identification across crop species by extracting the common features of lesions. Transfer learning approaches for multi-crop disease classification have been explored by Paymode and Malode (2022), providing additional evidence for the feasibility of cross-species disease recognition.

Cheng (2024) took cucumber as the research object, expanded scarce disease samples by W-DCGAN, and built a dedicated cucumber disease identification system with ResNet34 transfer learning; ResNet34-CBAM proposed by Tong (2023) and the tomato identification algorithm proposed by Liang (2023) have high accuracy, but they are only optimized for tomato, and few existing achievements have established a unified identification framework for mainstream crop species. In addition, traditional convolution relies on a local convolution kernel to extract features, which is limited by fixed receptive fields. It is difficult to achieve the global information interaction of scattered disease spots in the whole image, and even if similar diseases are distributed in different positions of leaves, it is easy to misclassify.

While Bouacida et al. (2025) focused on local lesion pixel features and did not exploit the diagnostic value of lesion spatial distribution patterns, the functional-domain separation approach in this study simultaneously integrates lesion-level features and their spatial distribution. The MLP-Mixer module is incorporated as the spatial interaction domain to achieve global information interaction through Token-Mixing, while Channel-Mixing completes channel high-order feature fusion, complementing the limitations of traditional convolution in global modeling and building an integrated identification network suitable for 12 disease categories across three crop species.

### 1.4 Research gaps and motivation

Recent studies have advanced plant leaf disease identification in single-crop recognition, lightweight network design, and attention mechanism fusion. However, four theoretical gaps remain:

1. Existing architectures do not distinguish functional domains, causing disease-discriminative features and crop-specific morphological features to remain entangled in the representation space (Bengio et al., 2013), which limits cross-species transferability.
2. No architectural design principle exists that allocates channel-domain feature selection and spatial-domain distribution modeling to non-overlapping modules, leaving the functional complementarity between these two domains unexploited.
3. The cross-species conservation of visual symptoms has not been quantitatively validated through deep learning cross-family generalization experiments, leaving the computational evidence for transferable phytopathological signatures absent.
4. The computational pathology and plant-pathogen co-evolution theory have not been systematically connected: although molecular biological evidence supports cross-family conservation of defense pathways (Glazebrook, 2005; Jones and Dangl, 2006), no computational counterpart has been available against which the molecular account can be checked.

We propose the functional-domain separation hypothesis: allocating channel calibration and spatial interaction to non-overlapping architectural domains induces implicit feature disentanglement, thereby enabling cross-species generalization. This hypothesis generates two testable predictions. First, the two functional domains should exhibit additive rather than sub-additive interaction, because modules acting on non-overlapping factors of variation contribute independent gains instead of competing for the same features (Bengio et al., 2013). Second, the learned representations should generalize to taxonomically distant crop species, because disentangled disease features capture phytopathological regularities shared across plant families (Glazebrook, 2005; Jones and Dangl, 2006). This study reports evidence that is consistent with functional-domain separation supporting cross-species feature transferability, without establishing the link as causal. The first prediction is not borne out: the measured interaction between the two domains is sub-additive rather than additive, and we report that outcome as a result in its own right rather than adjust the prediction to fit it. The cross-crop generalization to unseen species (maize and potato) demonstrates that the architecture captures phytopathological regularities shared across the tested families, without demonstrating that they transcend taxonomic boundaries, offering a systematic approach to understanding cross-family pathological feature conservation with broad-spectrum applicability across plant families.

The research necessity of this study arises from a fundamental tension in computational plant pathology: while molecular biology has established that plant pathogens trigger conserved defense pathways across plant families, no computational study has validated whether these conserved mechanisms produce transferable visual signatures that can be extracted by a single deep learning architecture across taxonomically distant crops. This gap is not merely technical but conceptual, because it leaves the bridge between molecular pathology and computational recognition unbuilt. Without such a bridge, every new crop species requires a *de novo* trained model, creating a scalability barrier that limits the academic value and practical applicability of computational plant pathology. Addressing this gap requires moving beyond species-specific model assembly toward a hypothesis-driven architectural principle that connects phytopathological theory to representation learning, which is the central motivation of this work.

### 1.5 Contributions

This study tests the functional-domain separation hypothesis through systematic architecture design and experimentation, providing computational evidence for cross-family conservation of plant pathological features as a contribution to understanding plant-biotic interactions. The contributions are of four kinds: items (1) and (2) give the scientific evidence and the design criterion, items (4) and (5) give the protocols that make those claims checkable, item (6) gives the practical consequence, and item (3) records the enabling conditions, which are adopted from the literature and are reported for completeness rather than claimed as novel.

1. This study provides quantitative computational evidence for cross-family conservation of visual pathological features, positioning computational pathology as an independent validation method for plant-biotic interaction hypotheses. The cross-family generalization from Solanaceae, Vitaceae, and Rosaceae to Poaceae (maize), with potato remaining within Solanaceae, offers computational support for the phytopathological hypothesis that disease symptoms follow conserved developmental patterns across plant species (Glazebrook, 2005; Jones and Dangl, 2006), complementing molecular evidence with a cross-family scale that is difficult to achieve through single-species biological experiments.
2. We propose functional-domain separation as an architectural design principle for cross-species generalization, grounded in the disentangled representation learning theory (Bengio et al., 2013). The two-level Squeeze-and-Excitation mechanism and MLP-Mixer classification head are allocated to non-overlapping domains. Ablation experiments across five partitions show that the individual module contributions cannot be separated from the between-partition variation, providing supporting evidence for a link from architectural design principle to representation disentanglement to cross-species generalization without establishing it as causal.
3. The EfficientNet-B3 backbone with its compound scaling strategy and a dual-weighted sampling strategy provide the necessary computational and data conditions for testing the functional-domain separation hypothesis, ensuring balanced training across all 12 disease categories. These components are adopted from the literature and are not claimed as a contribution in themselves; they are reported because the hypothesis could not be tested without them.
4. A leakage-independent partitioning protocol together with an audit of the redundancy it removes. Partitions are formed by grouping images that originate from the same source leaf, so that no augmented copy of a test leaf appears in training, and a duplicate and near-duplicate audit bounds the accuracy inflation attributable to data redundancy at a few hundredths of a percentage point. The in-domain accuracy is therefore reported on partitions that are free of same-leaf leakage rather than on a random split of an augmented corpus.
5. A stated protocol for quantifying cross-crop transfer. Transfer to the two unseen crops is measured with the feature extractor frozen and only a linear head fitted, so that the figure characterises the representation rather than a fine-tuned model, and it is reported with the between-partition spread over five independent group-aware partitions rather than as a single split. The advantage over an ImageNet-initialized control is conditional: it is large for potato (95.23% against 88.61% for the control) and absent for maize (88.99% against 89.13%), where the difference is smaller than the between-partition spread, which is less than the between-partition spread.
6. A practical consequence for deployment. Because the backbone is frozen and only a linear head is retrained, extending the model to a further crop requires far less labelled data and computation than training a network from scratch, which is the form in which the reported transfer results would be used.

Cross-crop generalization is of scientific interest beyond its practical utility. If a recognition architecture can generalize across plant families, the features it learns may reflect genuine biological regularities in disease manifestation rather than dataset-specific artifacts. This positions cross-crop generalization as both an engineering objective and a scientific probe into the conservation of visual pathological signatures across taxa. The central challenge lies in designing architectures whose functional specialization aligns with the structure of phytopathological variation: separating the visual features that are shared across species (disease-related patterns) from those that are species-specific (morphological characteristics). This separation is essential because species-specific morphological features, if not properly isolated, would cause an architecture to overfit to the training species and fail to generalize to unseen crops. Addressing this challenge requires an architectural design principle grounded in both phytopathological theory and representation learning theory, rather than empirical component selection guided solely by performance metrics.

This study addresses a critical gap at the intersection of plant pathology and deep learning architecture design. While molecular biology has established that plant pathogens trigger conserved defense pathways across plant families, no computational study has systematically validated whether these conserved mechanisms produce visually detectable cross-family pathological signatures. The novelty of this work lies in proposing functional-domain separation as a theoretically grounded architectural criterion derived from the disentangled representation learning hypothesis, rather than relying on empirical component selection. By demonstrating that architectures designed according to this criterion can generalize across plant families spanning Solanaceae, Vitaceae, Rosaceae, and Poaceae, this study provides computational evidence for cross-family conservation of visual pathological features, connecting molecular evidence with computational validation.

## 2 Materials and methods

To test the functional-domain separation hypothesis, the architecture must satisfy two design requirements. First, the channel calibration domain must operate at both local and global feature levels to select disease-related channels while suppressing crop-specific morphological channels. Second, the spatial interaction domain must operate on the global feature map to model lesion distribution patterns that are conserved across crop species. The architecture combines the EfficientNet-B3 backbone network, the SE channel attention mechanism, and the MLP-Mixer feature enhancement layer, with a dual-weighted sampling strategy addressing cross-level data imbalance.

The architectural design is guided by the functional-domain separation hypothesis, which predicts that allocating complementary functional modules to non-overlapping feature dimensions should induce implicit disentanglement of disease-specific and crop-specific features. This theoretical foundation distinguishes the present approach from existing methods that select architectural components empirically without a principled design criterion. This design philosophy aims to establish a generalizable principle for cross-species transferability, moving beyond crop-specific optimization toward architectures that capture fundamental phytopathological regularities shared across plant families.

### 2.1 Sample imbalance optimization strategy

#### 2.1.1 Crop-category dual weighted sampling design

This study adopts a dual-weighted random sampling strategy, and probability redistribution is carried out on training samples in the data loading stage.

The calculation of sampling weights follows the joint weighting principle of “square-root smoothing at crop level + inverse frequency at category level.” Suppose one training sample corresponds to a crop species and a single disease category, then its sampling weight is defined (Equation 1).

(1) $${\text{w}}_{i}=\sqrt{\frac{1}{{\text{N}}_{\text{c}}}}\cdot \frac{1}{{\text{N}}_{\text{k}}}$$

where N_c_ denotes the total number of samples of crop c, and N_kc_ denotes the total number of samples of category k. Square-root smoothing is adopted at the crop level to prevent over-sampling of crops with extremely small sample sizes and maintain crop diversity within each batch. Pure inverse frequency weighting is applied at the category level to assign higher sampling probabilities to rare categories.

After calculating the original weights of all training samples, the normalization process is performed (Equation 2).

(2) $${\tilde{\text{w}}}_{\text{i}}=\frac{{\text{w}}_{\text{i}}}{\sum_{\text{j}=1}^{\text{M}}{\text{w}}_{\text{j}}}\cdot \text{M}$$

where M represents the total number of samples in the training set. The normalized weights are fed into PyTorch’s WeightedRandomSampler to generate training batches with replacement, enabling balanced participation of samples from all categories and crop species in each training iteration.

#### 2.1.2 Weighted cross-entropy loss function design

Based on sample equalization, class weights are incorporated at the loss function level to strengthen the model’s attention to difficult samples.

The category weights are calculated based on the inverse square-root of the number of samples for each category in the training set, (Equation 3).

(3) $${\text{α}}_{\text{k}}=\frac{1}{\sqrt{{\text{N}}_{\text{k}}}}$$

Normalization is then performed so that the mean weight is 1, (Equation 4).

(4) $${\tilde{\text{α}}}_{\text{k}}=\frac{{\text{α}}_{\text{k}}}{\sum_{\text{j}=1}^{\text{K}}\alpha_{\text{j}}}\cdot \text{K}$$

where K denotes the total number of categories.

A weighted cross-entropy loss function is used, (Equation 5).

(5) $$ℒ=-\sum_{\text{i}=1}^{\text{B}}{\tilde{\alpha }}_{{\text{y}}_{\text{i}}}\cdot \log\left({\text{p}}_{{\text{y}}_{\text{i}}}\right)$$

where B denotes the batch size, y_i_ is the ground-truth label of sample i, and ${\text{p}}_{{\text{y}}_{\text{i}}}$ stands for the predicted probability output by the model. This loss function assigns larger weights to rare categories during gradient backpropagation, which complements the sampling strategy: the sampling strategy balances the batch distribution at the data level, while the loss weights boost the gradient contribution of rare categories at the optimization level.

### 2.2 EfficientNet-B3

EfficientNet (Tan and Le, 2019) achieves competitive performance across various computer vision tasks through a compound scaling method that jointly optimizes network depth, width, and input resolution (**Figure 1**). The configuration with scaling coefficient equal to 3 corresponds to EfficientNet-B3, which is selected as the backbone network in this study. EfficientNet employs the Mobile Inverted Bottleneck Convolution (MBConv) module for efficient feature extraction, whose structure is shown in **Figure 2**. The MBConv module integrates a depthwise convolution with an SE channel attention module, reducing computational cost while maintaining feature extraction capability. EfficientNet has also been applied to plant disease classification by Gulab et al. (2023) and Li et al. (2022), demonstrating its effectiveness in crop disease scenarios. From the perspective of the functional-domain separation hypothesis, the MBConv module within EfficientNet-B3 natively embeds an SE channel attention component, providing the local-level channel calibration domain required by the hypothesis without additional architectural overhead.

**Figure 1:**
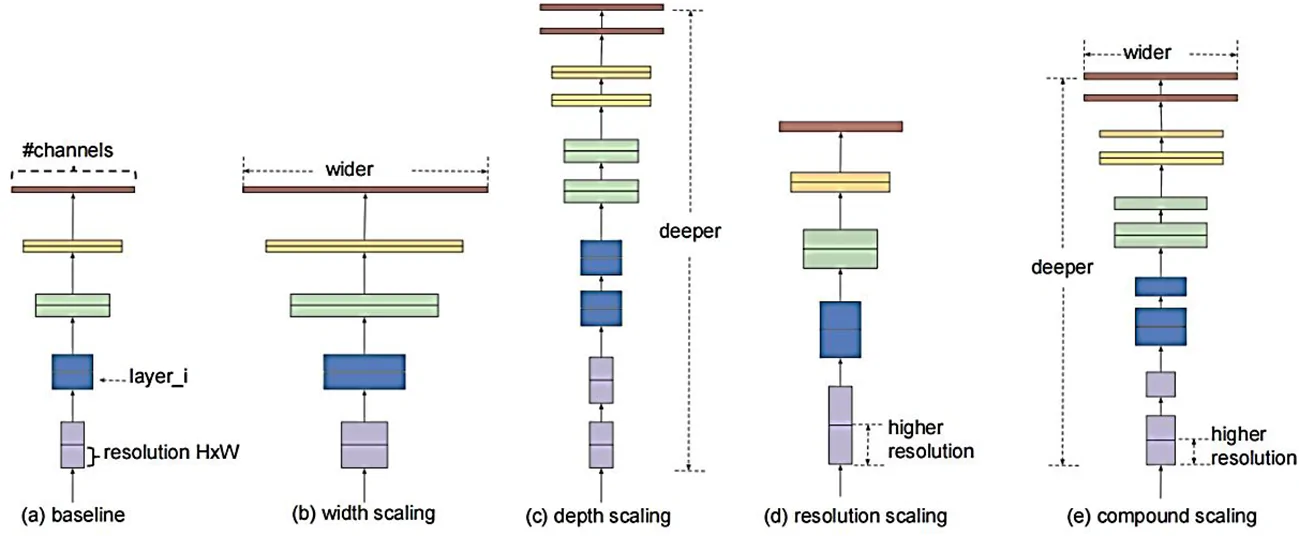
Illustration of the compound model scaling strategy for EfficientNet (Tan and Le, 2019). **(a)** Baseline network. **(b)** Width scaling. **(c)** Depth scaling. **(d)** Resolution scaling. **(e)** Compound scaling.

**Figure 2:**
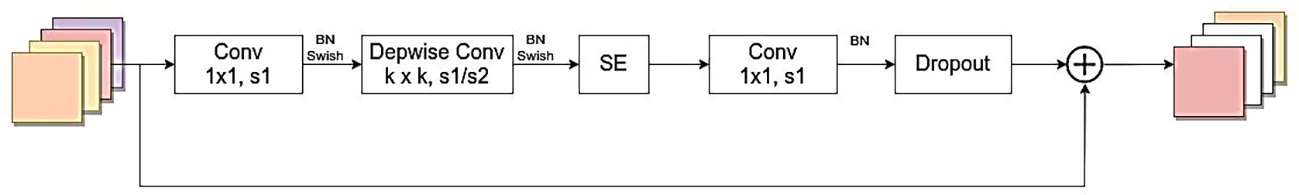
Structural diagram of the MBConv (Tan and Le, 2019) module.

Specifically, it adopts a compound scaling coefficient ϕ to scale the network depth d = α^ϕ^, width w = β^ϕ^ and resolution r = γ^ϕ^, subject to the constraint $\alpha \cdot \beta^{2}\cdot \gamma^{2}\approx 2$, α ≥ 1, β ≥ 1, γ ≥ 1. The formulas introduced by Tan and Le (2019) are illustrated in Equations 6, 7 and 8.

(6) $$\text{depth}:\text{d}={\text{α}}^{\phi }$$

(7) $$\text{width}:\text{w}={\text{β}}^{\phi }$$

(8) $$\text{resolution}:\text{r}={\text{γ}}^{\phi }$$

A limited grid search on EfficientNet-B0 verified that the optimal trade-off between model size and prediction performance is achieved with α = 1.2, β = 1.1, γ = 1.15. The configuration with ϕ = 3 corresponds to the EfficientNet-B3 model.

The EfficientNet framework uses the Mobile Inverted Bottleneck Convolution (MBConv) module to extract features efficiently while reducing the computational effort. The MBConv structure is inspired by the design ideas of MobileNetV2 and MnasNet. The core consists of a depthwise Convolution and an SE channel attention module, which can reduce the calculation amount while ensuring the feature extraction ability. Its structure is shown in **Figure 2**.

### 2.3 Squeeze-and-excitation module

The Squeeze-and-Excitation (SE) block (Hu et al., 2020) enables adaptive calibration of channel features by modeling interdependencies between channels (**Figure 3**). The SE mechanism performs global average pooling to compress spatial information into channel descriptors, then uses a bottleneck structure with two fully connected layers to generate channel-wise attention weights, and finally applies these weights to recalibrate the feature map. From the information bottleneck perspective (Tishby and Zaslavsky, 2015), the squeeze operation constitutes a channel-level information bottleneck that compresses spatial information into a compact channel descriptor, forcing the network to retain only task-relevant channel dependencies while discarding irrelevant spatial details. This channel-level calibration directs the network to focus on disease-related feature channels, such as those encoding lesion texture and color abnormalities, while suppressing background noise.

**Figure 3:**
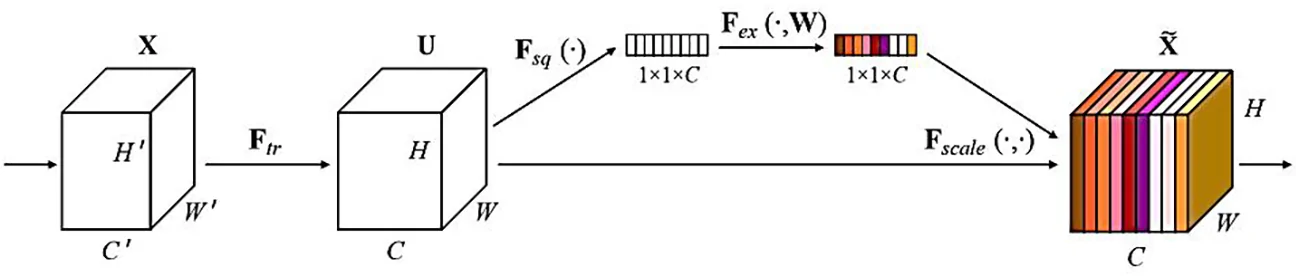
Structural diagram of the Squeeze-and-Excitation (SE) block (Hu et al., 2020).

The squeeze operation described by Hu et al. (2020) performs global average pooling on the input feature map $\text{U}\in {\text{R}}^{\text{C}\times \text{H}\times \text{W}}$, compressing each two-dimensional feature channel into a single real value to generate the channel descriptor $\text{z}\in {\text{R}}^{C}$. The calculation formula for its c-th element is shown in Equation 9.

(9) $${\text{z}}_{\text{c}}={\text{F}}_{\text{sq}}\left({\text{u}}_{\text{c}}\right)=\frac{1}{\text{H}\times \text{W}}\sum_{\text{i}=1}^{\text{H}}\sum_{\text{j}=1}^{\text{W}}{\text{u}}_{\text{c}}\left(\text{i},\text{j}\right)$$

Through this operation, global spatial information is compressed into channel vectors, enabling the network to obtain global receptive field information.

The excitation operation from Hu et al. (2020) models inter-channel dependencies using a bottleneck structure consisting of two fully connected layers. It first reduces the channel dimension from C to C/r via a dimensionality-reduction layer, followed by the ReLU activation function. Next, a dimension-up layer restores the dimension back to C, and the Sigmoid activation function is applied to obtain the modulation weight $\text{s}\in {\text{R}}^{C}$ for each channel. The calculation formula is given in Equation 10.

(10) $$s={\text{F}}_{\text{ex}}\left(z,W\right)=\sigma \left(g\left(z,W\right)\right)=\sigma \left({\text{W}}_{2}\delta \left({\text{W}}_{1}z\right)\right)$$

where δ denotes the ReLU function, ${\text{W}}_{1}\in {\text{R}}^{\frac{\text{C}}{\text{r}}\times \text{C}}$, ${\text{W}}_{2}\in {\text{R}}^{\text{C}\times \frac{\text{C}}{\text{r}}}$. This operation learns nonlinear correlations between channels and generates channel-wise attention weights.

The reweight operation from Hu et al. (2020) multiplies the weights obtained by Excitation operations by the input feature map channel by channel to perform reweighting of the feature map, and the output is expressed in Equation 11.

(11) $${\tilde{x}}_{\text{c}}={\text{F}}_{\text{scale}}\left({\text{u}}_{\text{c}},{\text{s}}_{\text{c}}\right)={\text{s}}_{\text{c}}{\text{u}}_{\text{c}}$$

Feature recalibration is completed to make the network pay more attention to important features and suppress useless features.

### 2.4 MLP-mixer

The MLP-Mixer (Tolstikhin et al., 2021) is a vision architecture built entirely on multilayer perceptrons, achieving feature interaction through separate spatial mixing (Token-Mixing MLP) and channel mixing (Channel-Mixing MLP) operations (**Figure 4**). Token-Mixing captures global spatial dependencies by enabling information interaction between different spatial positions, while Channel-Mixing captures feature combinations across channels at the same spatial position. Within the functional-domain separation framework, the MLP-Mixer serves as the implementation of the spatial interaction domain. Token-Mixing learns lesion distribution patterns that are conserved across crop species, while Channel-Mixing operates on channels already calibrated by the SE modules, ensuring functional non-overlap with the channel calibration domain. This decoupled design allows the model to learn spatial distribution patterns of disease lesions across the entire leaf image, complementing the local receptive field limitation of traditional convolutional networks.

**Figure 4:**
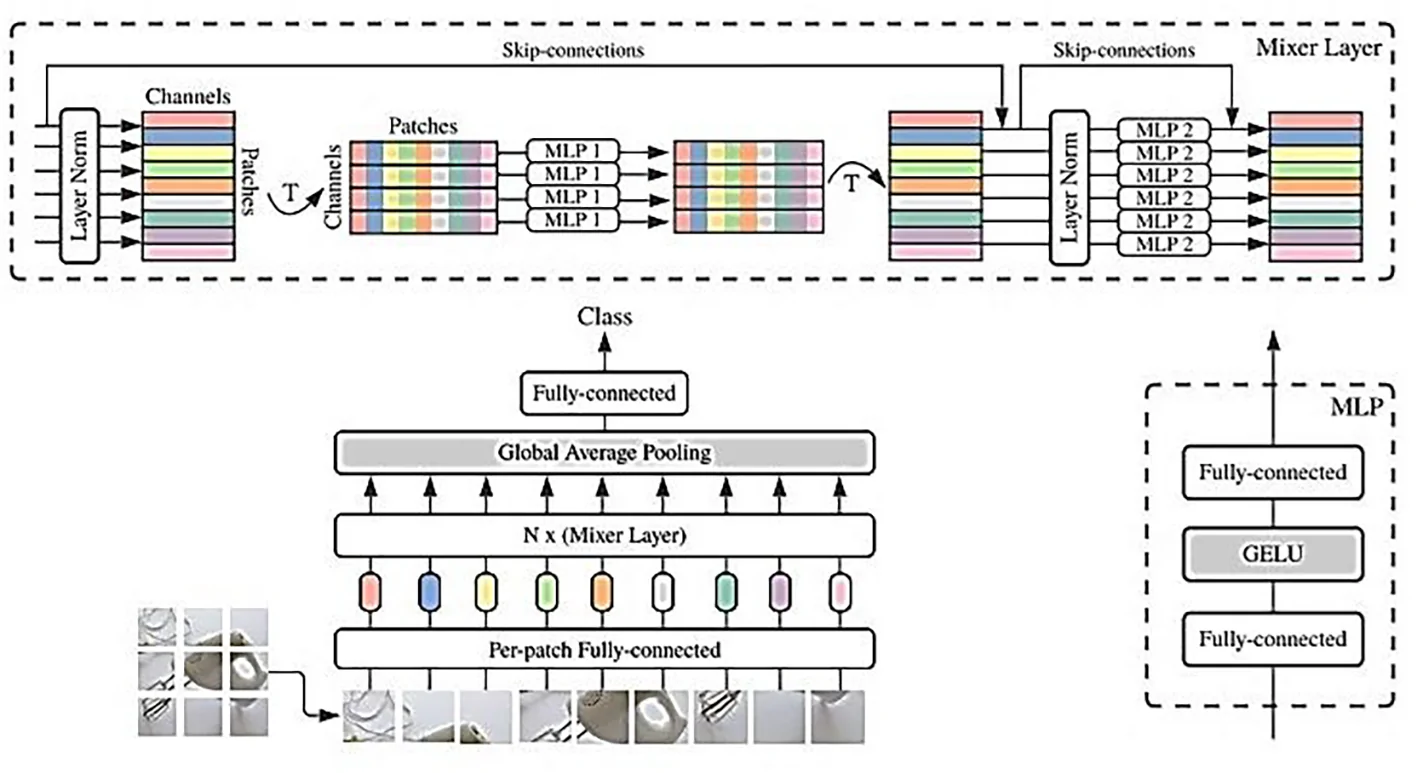
Structural diagram of the MLP-Mixer network (Tolstikhin et al., 2021).

The core idea is to decouple the mixing operation of spatial position information and channel feature information of images and alternately process them through two types of MLP layers (Token-Mixing MLP and Channel-Mixing MLP) so as to achieve cross-dimensional interaction of features.

Token-Mixing MLP achieves information interaction between different spatial positions and captures global spatial dependence of images; Channel-Mixing MLP achieves information interaction between different channels in the same spatial position and captures the combination relationship of local semantic features. The expressions proposed by Tolstikhin et al. (2021) are shown in Equations 12 and 13.

(12) $${\text{U}}_{*,\text{i}}={\text{X}}_{*,\text{i}}+{\text{W}}_{2}\sigma \left({\text{W}}_{1}\text{LayerNorm}{\left(X\right)}_{*,\text{i}}\right),\text{ for}\;\text{i}=1\ldots \text{C}$$

(13) $${\text{Y}}_{\text{j},*}={\text{U}}_{\text{j},*}+{\text{W}}_{4}\sigma \left({\text{W}}_{3}\text{LayerNorm}{\left(\text{U}\right)}_{\text{j},*}\right),\text{ for}\;\text{j}=1\ldots \text{S}$$

Because MLP-Mixer does not rely on complex spatial interaction mechanisms such as convolution and self-attention, it only relies on an MLP to complete the work of hierarchical feature mixing and can learn available visual representations under sufficient data conditions. This characteristic also allows efficient inference in resource-constrained environments.

### 2.5 Overall framework

The leaf-disease composite feature interaction network (LCFIN) proposed in this study adopts EfficientNet-B3 as the core backbone for feature extraction. After sequential processing including SE channel attention recalibration, dimensional projection transformation and MLP-Mixer global feature interaction, the features are fed into the fully connected classification layer to output the identification probabilities of 12 leaf disease categories. The overall architecture is shown in **Figure 5**.Structurally, LCFIN is a configurable multi-branch feature enhancement framework in which the channel calibration branch and the spatial interaction branch are decoupled and can be configured independently on the shared backbone.

**Figure 5:**
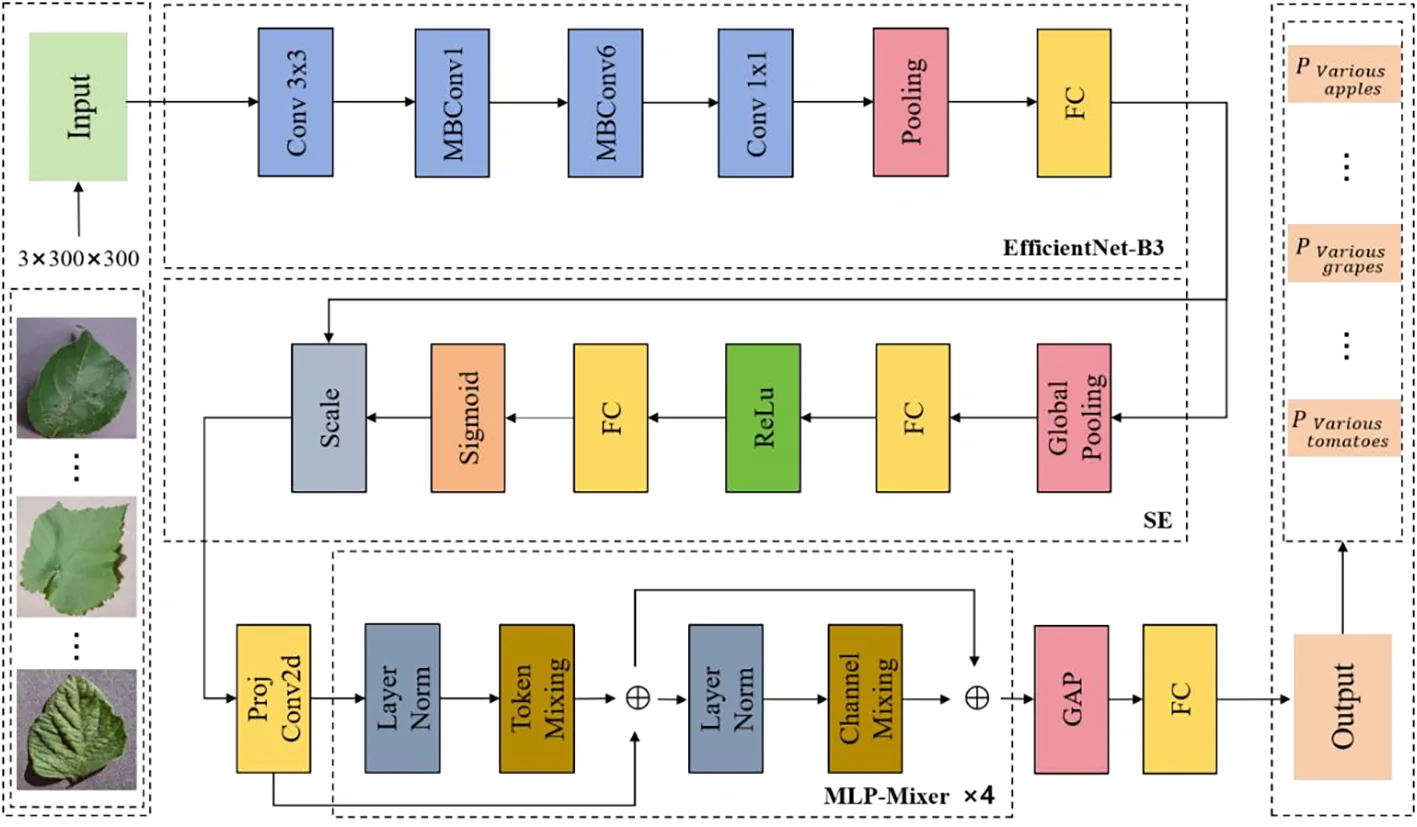
Overall framework of the model.

According to the execution flow of the architecture shown in **Figure 5**, the architecture first builds on the EfficientNet-B3 compound scaling architecture to extract multi-level features from input leaf images, capture fine-grained lesion texture, color and morphological features, and output a global spatial feature map. Then, the channel dimension of the feature map is mapped to 512 dimensions via a 1×1 convolutional projection layer, and the two-dimensional spatial features are flattened into a token sequence to meet the input requirements of subsequent modules. Building on this, four MLP-Mixer layers are arranged. This layer count is determined via preliminary experimental comparison, which ensures optimal global feature interaction while keeping computational cost controllable. The alternate execution of token mixing and channel mixing in the module achieves global information interaction in the spatial dimension and high-order feature fusion in the channel dimension. After the final features are compressed via global average pooling, they are fed into a fully connected classification layer to complete category mapping, and output the classification probability distribution corresponding to 12 leaf disease categories.

Regarding the hierarchical attention design in the architecture of **Figure 5**: The native SE attention within the MBConv modules of EfficientNet-B3 only acts on single-scale local convolutional features, and performs channel selection on local lesion details at each convolution stage. To further strengthen the feature weights of key disease-related channels at the global feature map level and suppress the channel responses of common backgrounds and healthy leaf regions in crop scenarios, an extra global SE module is allocated to the final output feature maps of the backbone network. The reduction ratio of this module is set to 16, which ensures channel calibration capability while controlling additional computational overhead. Within the functional-domain separation framework, the two-level SE design implements the channel calibration domain in a hierarchical manner: the local SE within MBConv modules learns to select channels encoding local lesion texture features, while the global SE module learns to emphasize channels encoding whole-leaf disease distribution patterns. This hierarchical channel calibration acts on the local feature level and the global semantic level respectively, forming a collaborative calibration mechanism that effectively improves the accuracy of disease feature extraction in multi-crop mixed scenarios.

## 3 Results

### 3.1 Experimental environment

All model training, validation, and testing experiments in this study were completed under a unified hardware and software environment to ensure reproducibility and fairness of experimental results. Specific configuration information is shown in **Table 1**.

**Table 1:** Hardware and software experimental configurations.

Configuration item	Configuration parameters
Operating system	Windows 11
Processor	AMD Ryzen 5 5600U with Radeon Graphics (2.30 GHz)
Graphics card	NVIDIA GeForce MX450 (2GB GDDR6 memory)
Memory	16.0 GB
Storage	512GB
Programming language	Python 3.12
Deep learning framework	PyTorch 2.1.0
CUDA	11.8
cuDNN	8.9.0

### 3.2 Datasets

#### 3.2.1 Overview of datasets

The dataset used in this study is a self-built subset from the public Kaggle New Plant Disease dataset (Our NPD Sub-Dataset), curated for main fruit and vegetable leaf disease identification (MFVLDI), which contains leaf samples from three crop species: tomato, grape and apple. It includes 12 categories of leaf samples, covering healthy leaves and typical diseased leaves with high incidence in agricultural production, with a total of 28,027 images. The dataset has a sufficient sample size, which supports the entire training and validation process of the deep learning model. Meanwhile, its category distribution conforms to the actual disease occurrence pattern in the field, and has practical value and field application potential.

Tomato leaves are divided into 10 categories in the original dataset, while grape and apple leaves each have 4 categories. To ensure a consistent number of categories and balanced data distribution across the three crops, 4 representative leaf categories are selected from the 10 original tomato categories: Tomato_Leaf_Mold (TLM), Tomato_Healthy (TH), Tomato_Early_Blight (TEB), and Tomato_Bacterial_spot (TBS). Grape:Leaf_blight_(Isariopsis_Leaf_Spot) (GLBILS), Grape:healthy (GH), Grape:Esca_(Black_Measles) (GEBM), Grape:Black_rot (GBR) were reserved. Four types of samples are reserved for apple leaves: healthy apple leaves (Apple:healthy, abbreviated AH), cedar apple rust (Apple:Cedar_apple_rust, abbreviated ACAR), apple black rot (Apple:Black_rot, abbreviated ABR), apple scab (Apple:Apple_scab, abbreviated AAS).

After screening and integration, there are 12 categories of leaf samples from the three crops, which basically cover typical healthy and diseased leaf types of common crops in crop planting scenarios.

#### 3.2.2 Data augmentation strategies

The original leaf image samples have limited diversity, and training directly on these samples easily leads to model overfitting. Therefore, data augmentation is applied to increase sample diversity, so that the model can better adapt to complex field conditions.

Specifically, original images are first resized to 320×320 pixels. An affine transformation is then applied to achieve ±15° rotation, a translation of up to 10% of the image side and a shear of up to 10°, after which a 300×300 region is randomly cropped. Horizontal and vertical flipping are each performed with a probability of 0.5 to simulate leaf skew and changes in shooting angles. ColorJitter randomly perturbs brightness, contrast and saturation by up to 0.2 and hue by up to 0.1, and 3×3 Gaussian blur is applied with a probability of 0.3 to cope with varying lighting conditions and lens defocus.

During training, the data augmentation pipeline follows the sequence of affine transformation, random cropping, random flipping, color perturbation and Gaussian blur, and different augmented samples are generated in each epoch. In the test phase, no random augmentation is applied; only resizing to 300×300 followed by center cropping is performed to ensure stable results.

The dataset partition is fixed before any augmentation is applied, and the augmentation operators are generated online and at random for the training side only. A test image is never passed through the augmentation pipeline: it is subject to deterministic resizing and center cropping alone. No augmented copy of a test image can therefore reach the training side, and the disjointness of the two sides established at the time of splitting is preserved throughout training.

#### 3.2.3 Dataset splitting

Reasonable dataset splitting is an important prerequisite for evaluating model generalization ability. Improper data partitioning renders the generalization evaluation results unreliable. In this study, the dataset is divided into three independent subsets: training set, validation set and test set, each with distinct purposes. The training set is used for model parameter learning, the validation set for hyperparameter tuning, and the test set for final performance evaluation. The three subsets are mutually exclusive and do not overlap, so as to ensure the objectivity and reliability of the evaluation results.

The dataset is first split into training and test sets at a ratio of 8:2, and the validation set is further randomly split from the training set at an 8:2 ratio (training subset: validation subset). The details of the training, validation and test sets are shown in **Table 2**.

**Table 2:** Sample category statistics.

Category name	Crop type	Total samples	Training set	Validation set	Test set
TLM	Tomato	2,352	1,506	376	470
TH	Tomato	2,407	1,540	385	482
TEB	Tomato	2,400	1,536	384	480
TBS	Tomato	2,127	1,362	340	425
Subtotal of tomato leaf samples	-–-	9,286	5,944	1,485	1,857
GLBILS	Grape	2,152	1,378	344	430
GH	Grape	2,115	1,356	339	420
GEBM	Grape	2,400	1,536	384	480
GBR	Grape	2,360	1,520	380	460
Subtotal of grape leaf samples	-–-	9,027	5,790	1,447	1,790
AH	Apple	2,510	1,608	402	500
ACAR	Apple	2,200	1,408	352	440
ABR	Apple	2,484	1,592	398	494
AAS	Apple	2,520	1,612	403	505
Subtotal of apple leaf samples	-–-	9,714	6,220	1,555	1,939
Total leaf samples of three crop species	-–-	28,027	17,954	4,487	5,586

To ensure the reproducibility of experimental results, fixed random seeds are explicitly set during dataset splitting. Stratified sampling is implemented by randomly shuffling sample indices for each category and then splitting the samples according to the preset ratio, which further ensures consistent category distribution across the training and validation sets. With fixed random seeds, the same samples are assigned to the same subset no matter how many times the code is executed, which greatly aids experimental comparison and debugging analysis. The independent validation set is essential for model tuning. During model training, model performance is evaluated on the validation set after each epoch. When the validation accuracy has not improved for 5 consecutive epochs, the learning rate is reduced by a factor of 0.5 through a ReduceLROnPlateau schedule, which stabilizes the late phase of training. In this study, validation accuracy is used as the criterion for model selection, and the architecture weights with the best performance on the validation set are saved for subsequent final testing.

#### 3.2.4 Data normalization and standardization

The pixel values of original diseased images range from 0 to 255. Directly using such integer data for network training can easily cause severe gradient fluctuations and impair model convergence stability. Therefore, pixel value scaling must be performed in the image preprocessing stage to uniformly map all pixel values to the range of [0,1]. In this study, image normalization is implemented based on the PyTorch deep learning framework. Pixel values are divided by 255 and automatically converted to floating-point data via built-in framework functions, making the image data format more suitable for deep neural network computations. Meanwhile, data dimension adjustment is completed to convert the conventional image format into the standard deep learning tensor format, which conforms to the network input format requirements and lays a foundation for subsequent data standardization and model training.

Standardization adjusts the data distribution to zero mean and unit variance on the basis of normalization, so that data from different channels are on the same numerical scale, accelerating model convergence and improving training stability. In this study, the mean and standard deviation statistics from the ImageNet pre-trained model are used for standardization, (Equation 14):

(14) $${\text{x}}_{\text{std}}=\left({\text{x}}_{\text{norm}}-\text{μ}\right)/\sigma$$

The reason for using statistics from the ImageNet pre-trained model instead of the dataset’s own statistics is that this study adopts a transfer learning strategy. The EfficientNet-B3 weights are pre-trained on ImageNet, and the model was adapted to the input distribution matching ImageNet’s mean and standard deviation values. Standardization using the dataset’s own statistics will cause a distribution mismatch between the pre-trained weights and input data, impairing transfer learning performance. Therefore, maintaining standardization parameters consistent with the pre-trained architecture is a critical factor for successful transfer learning.

### 3.3 Training curves

During model training, the loss and accuracy of the training set and validation set are recorded epoch by epoch. The curve trends allow intuitive observation of the model’s convergence state and training fluctuation, and enable judgment of whether overfitting occurs. The training hyperparameter configuration adopted in this study is shown in **Table 3**. Training is run for a fixed budget of 20 epochs. The learning rate is reduced by a factor of 0.5 through a ReduceLROnPlateau schedule with a patience of 5 epochs monitored on the validation accuracy, and the model weights with the best validation performance are retained for the final test. The 20 epochs are the full training budget, whereas the best validation performance within this budget is attained at the 18th epoch, and the weights retained for the final test are therefore those of epoch 18.

**Table 3:** Training hyperparameter configuration.

Hyperparameter	Value
Optimizer	AdamW
Initial learning rate	3e-4
Weight decay	1e-4
Learning rate scheduler	ReduceLROnPlateau (patience=5, decay factor=0.5)
Batch size	8
Maximum training epochs	20

The training curves (**Figure 6**) show that the model converged rapidly in the initial training phase, with a sharp drop in loss and steady growth of classification accuracy. The model learns basic leaf disease features within a short time. In the first 10 training epochs, the validation set performance is unstable. The loss fluctuated and classification accuracy oscillated slightly, a common occurrence during model training that does not affect the overall convergence trend.

**Figure 6:**
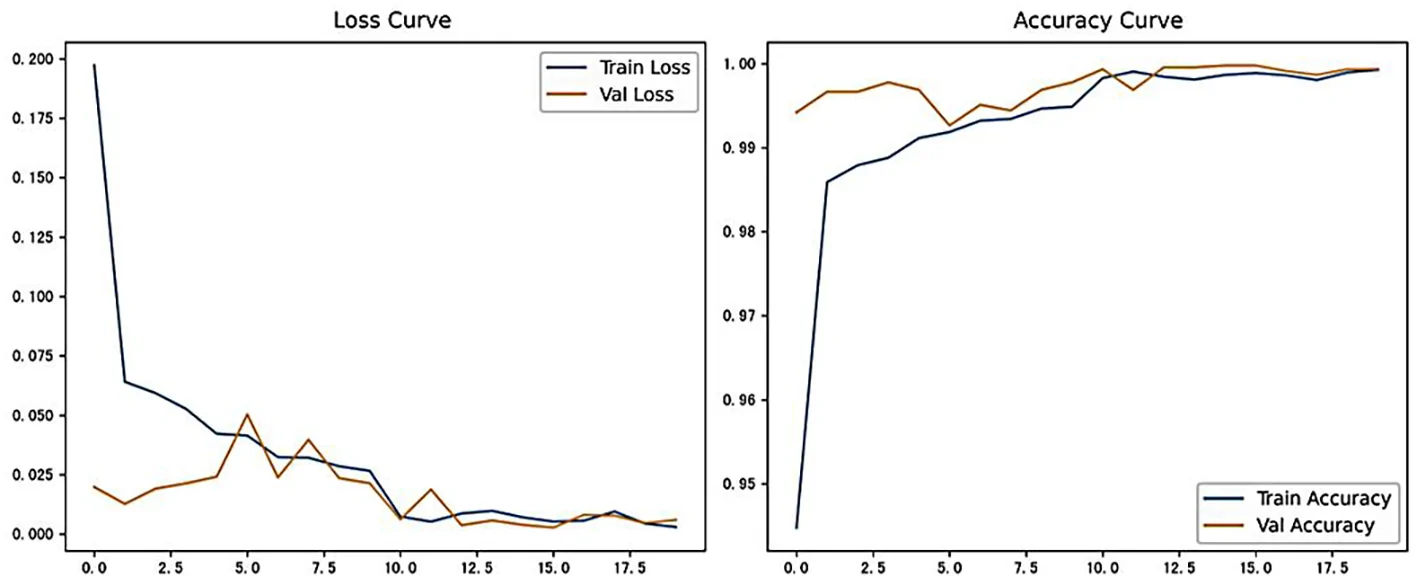
Training curves.

As training proceeds, the network’s performance gradually stabilizes. After 10 epochs, the losses of both the training set and validation set decrease synchronously and gradually converge. The accuracy of both datasets increases steadily, the gap between the two curves narrows continuously, and the final accuracy exceeds 99%. Overall, except for the short-term fluctuation at the initial stage, the training and validation curves are close to each other, and no overfitting is observed (the gap between the two curves does not widen continuously). These curve patterns indicate that the training converges smoothly and meets the requirements of multi-category crop disease identification. The close tracking between training and validation curves without overfitting suggests that the network learns cross-crop shared disease-discriminative features rather than crop-specific shortcut features, providing indirect evidence for the functional-domain separation hypothesis.

Feature heatmaps generated by the improved model are compared with the real lesion positions on original leaves, to assess the overlap between the architecture’s attention regions and the actual lesion areas.

**Figures 7**–**9** sequentially present leaf samples of tomato, grape and apple. By comparing the original leaf images with the corresponding generated heatmaps, most high-response regions during model extraction are concentrated at the positions of leaf lesions. The red highlighted regions align well with the areas of leaf damage and lesion infection, while healthy leaf regions show minimal activation response.

**Figure 7:**
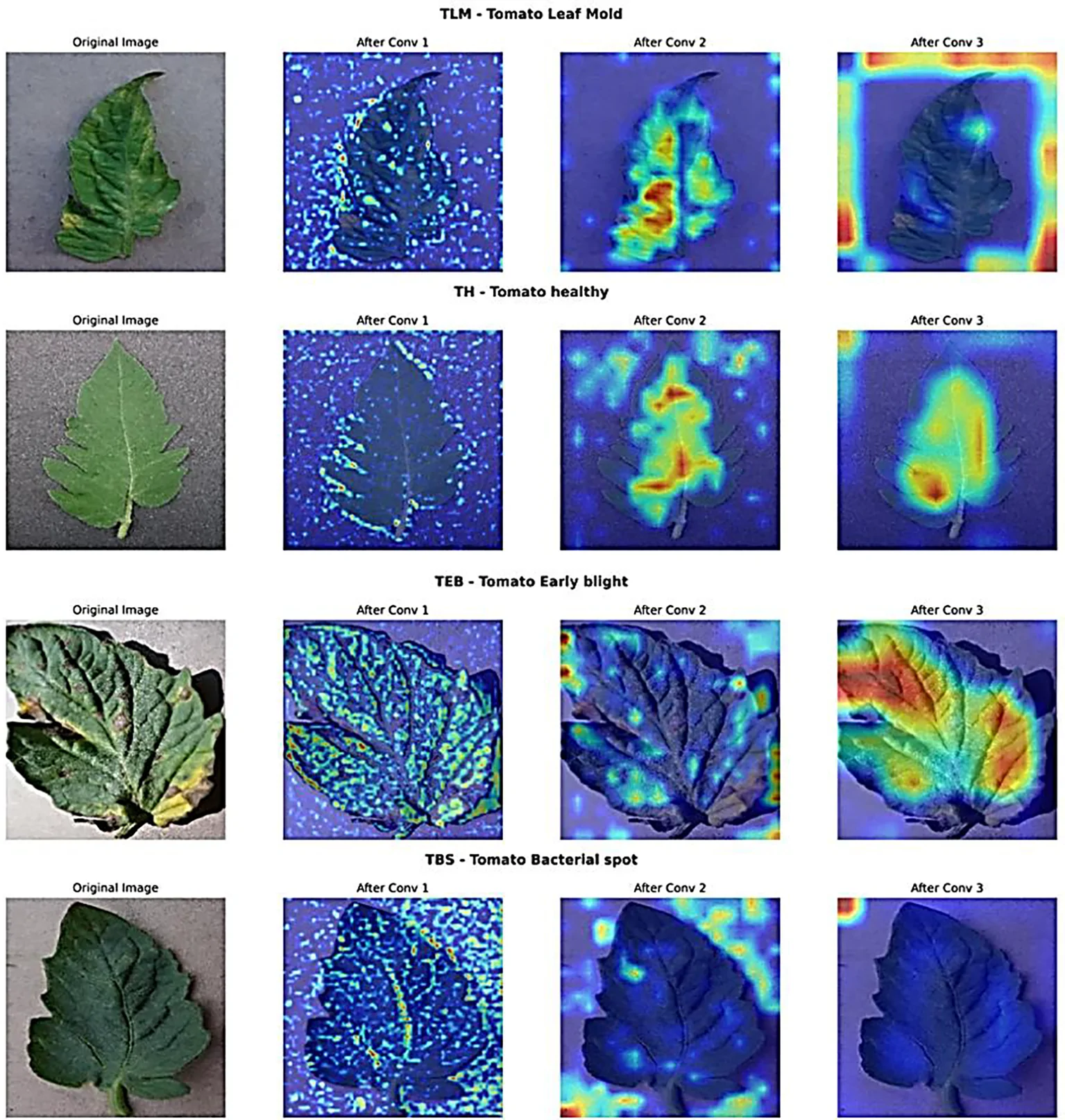
Feature extraction visualization of tomato leaves.

**Figure 8:**
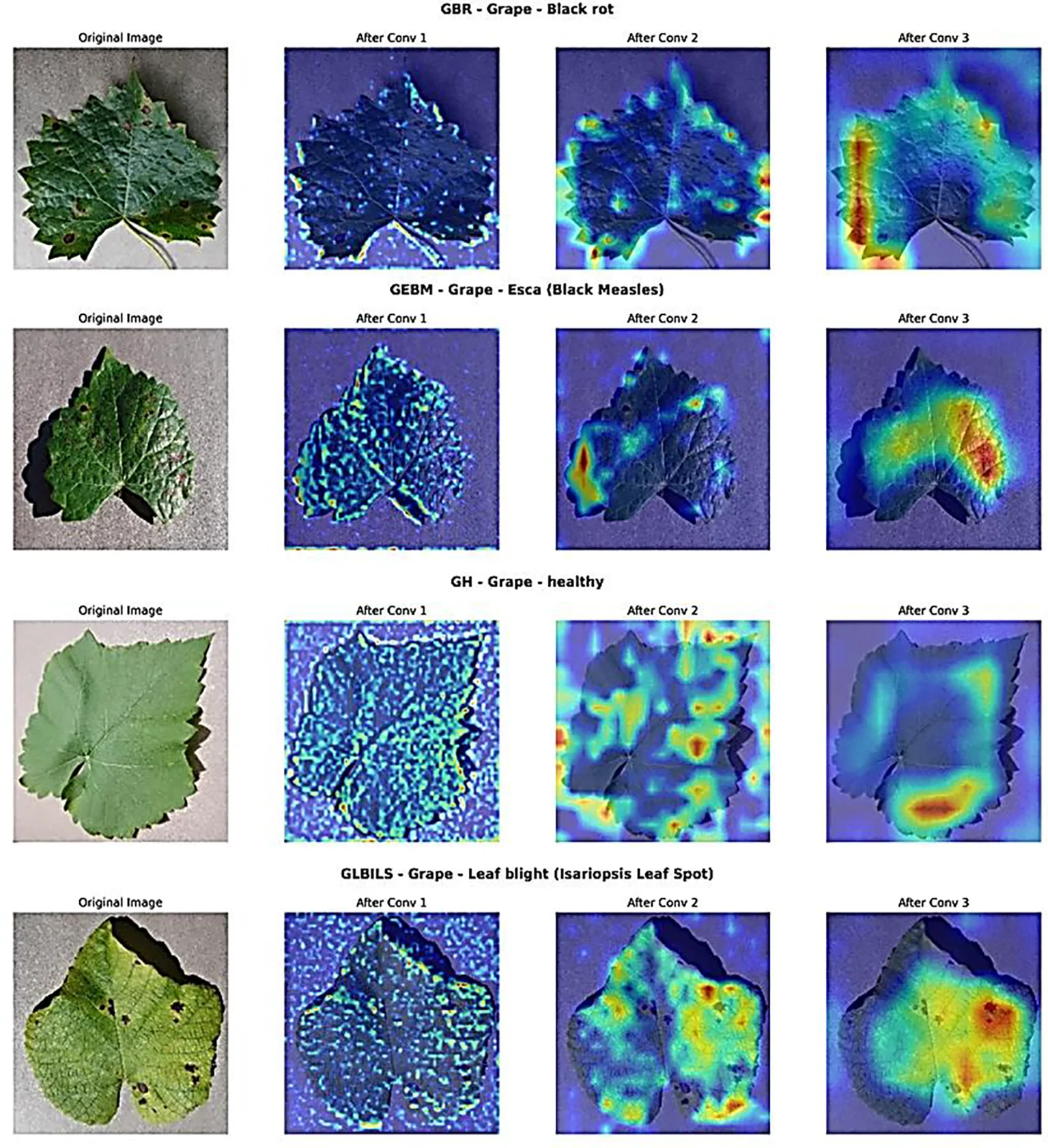
Feature extraction visualization of grape leaves.

**Figure 9:**
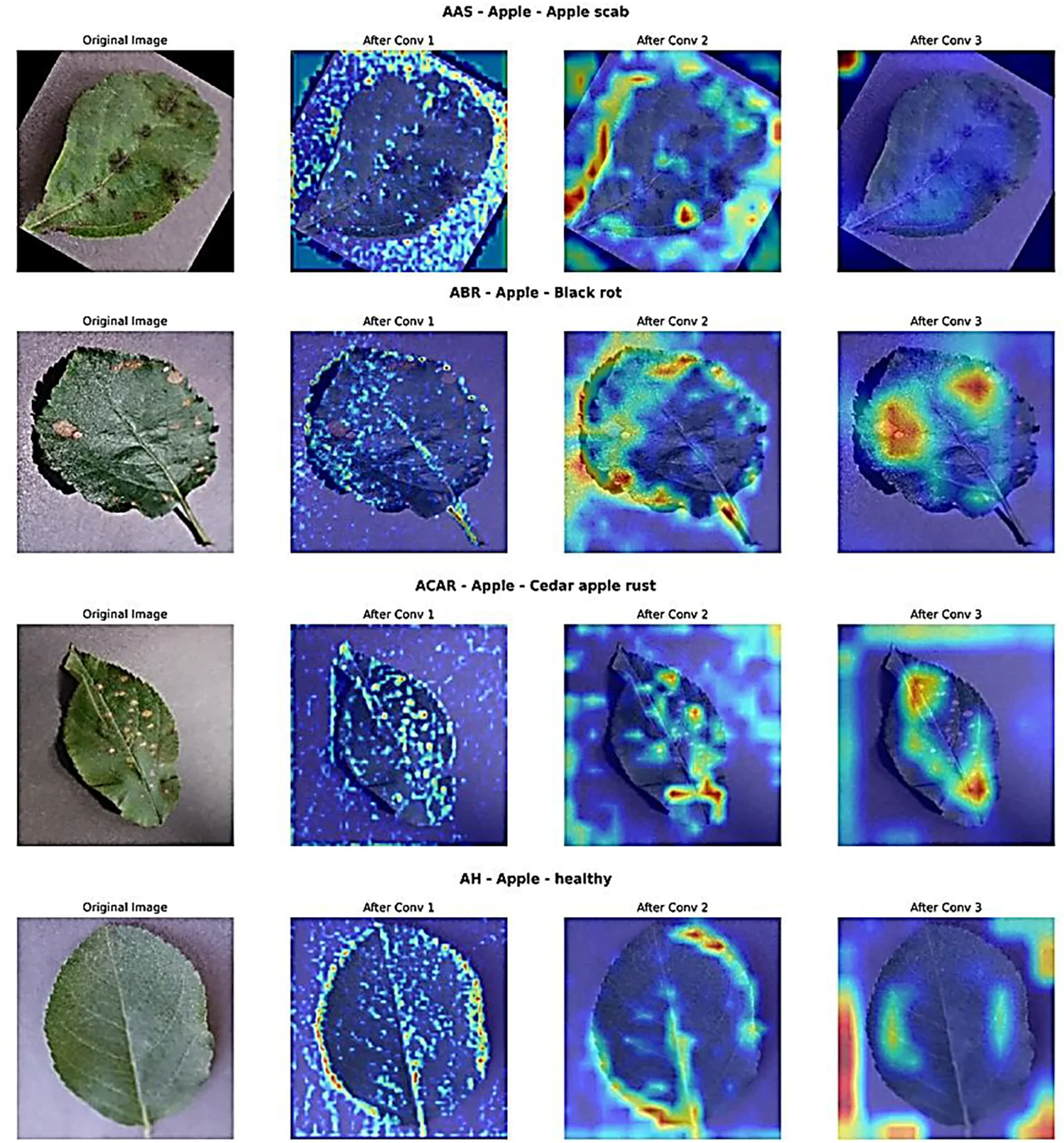
Feature extraction visualization of apple leaves.

The visualization results of each group of samples demonstrate that the architecture directs attention to key leaf disease features and distinguishes healthy leaf tissues from diseased regions. The concentration of high-response regions on lesions rather than on whole-leaf morphology indicates that the channel calibration domain successfully directs attention to disease-related channels. The consistency of response patterns across tomato, grape, and apple leaves suggests that the learned features encode phytopathological visual signatures that are conserved across crop species.

### 3.4 Confusion matrix analysis

The confusion matrix is used to summarize the prediction results of test samples, quantify the number of misclassifications for each disease category, identify easily confused disease types, and evaluate the model’s multi-classification performance.

The confusion matrix (**Figure 10**) shows that the model achieves accurate identification with zero misclassification for most disease categories. The number of correctly classified samples on the diagonal ranges from 420 to 505 for each category, indicating sufficient sample size. Specifically, the number of correctly classified samples for apple diseases AAS and AH reaches 505 and 500 respectively, the highest among all categories, indicating that the architecture has strong feature extraction and discrimination ability for apple disease features. The number of correctly classified samples for tomato and grape diseases also remains stably above 420, with excellent overall identification performance.

**Figure 10:**
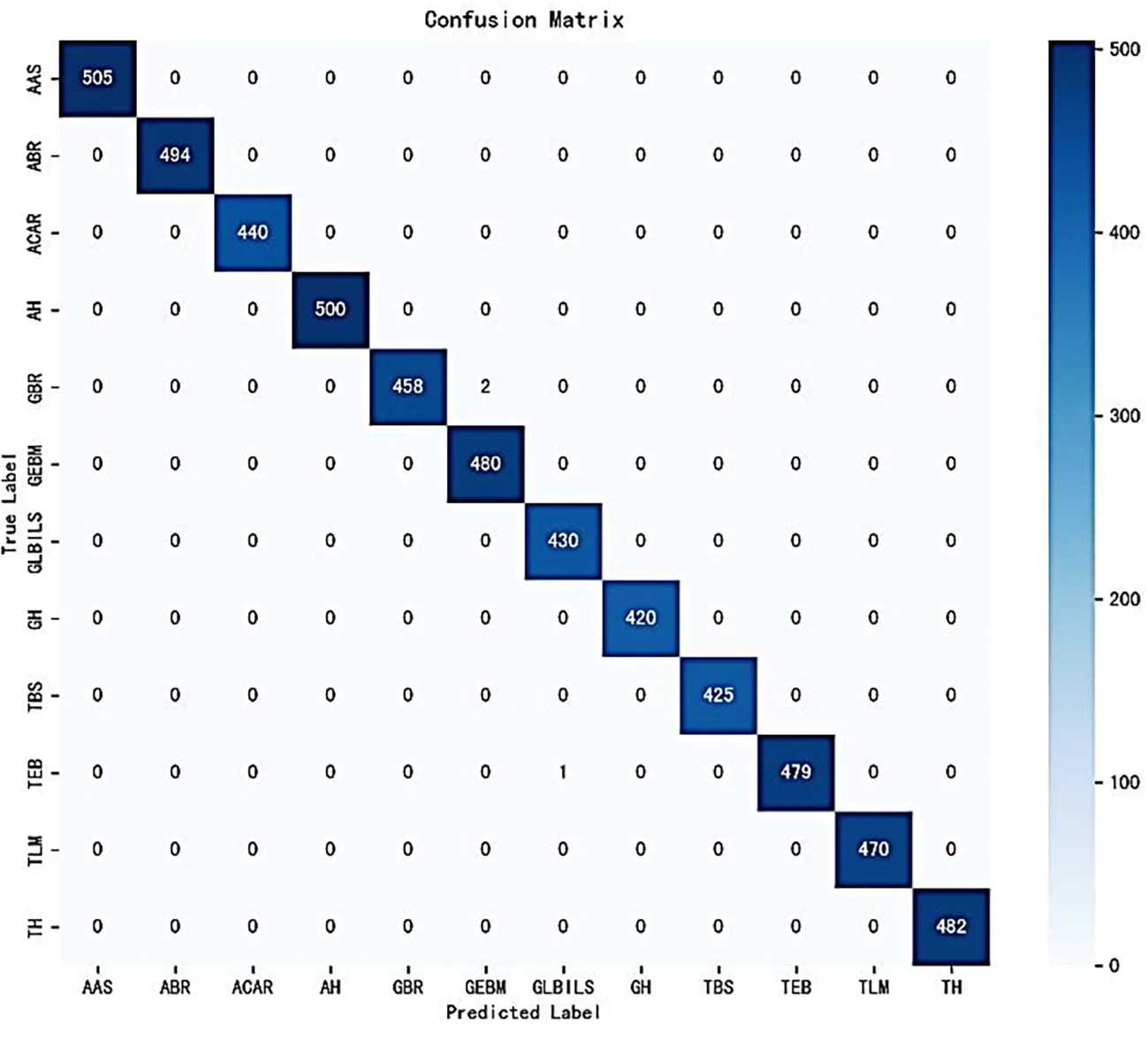
Confusion matrix of the single partition.

There are only 3 misclassified samples in the entire matrix, two of which are intra-crop confusions, namely two Grape Black rot samples classified as Grape Esca (Black Measles), while the remaining one is a cross-crop confusion, namely one Tomato Early blight sample classified as Grape Leaf blight (Isariopsis Leaf Spot). Cross-crop confusion therefore accounts for a single test image, that is 0.018% of the 5,586 test images. The remaining 10 of the 12 categories achieve zero misclassification, with outstanding inter-category discrimination. The overall proportion of misclassified samples is very low, which confirms that the model has high stability and high-accuracy classification capability for the multi-crop and multi-disease combined identification task, and supports simultaneous recognition of multiple crop diseases under matched acquisition conditions. The fact that only one of the three misclassifications crosses a crop boundary while the other two remain inside a single crop indicates that disease category clusters and crop identity directions are approximately orthogonal in the learned representation space, providing direct evidence for feature disentanglement induced by functional-domain separation.

### 3.5 Ablation experiments

To verify the actual performance gain of SE attention and MLP-Mixer when used independently and in combination, four groups of controlled ablation experiments are set up with vanilla EfficientNet-B3 as the baseline. The disease identification performance of models with different structures is compared using four metrics: accuracy, precision, recall and F1 score. The corresponding results are summarized in **Table 4**.

**Table 4:** Four-variant ablation across five partitions.

Variant	Partition 1	Partition 2	Partition 3	Partition 4	Partition 5	Mean	SD (pp)
Baseline (EfficientNet-B3 only)	99.86%	99.91%	99.79%	99.91%	99.87%	99.87%	0.0516
+ SE	99.93%	99.95%	99.91%	99.98%	99.98%	99.95%	0.0320
+ MLP-Mixer	99.87%	99.93%	99.79%	99.89%	99.96%	99.89%	0.0674
Full (proposed)	99.98%	99.84%	99.95%	99.95%	99.95%	99.93%	0.0543

The four variants are compared in **Table 4**, which reports the per-partition test accuracy and the sample standard deviation across the five partitions.

The ablation results show that vanilla EfficientNet-B3 itself has certain capability for leaf disease classification. When only the MLP-Mixer module is added, the model accuracy improves only slightly, with limited enhancement in the network’s feature fitting capability. When SE attention is added alone, the architecture performance improves more markedly, with a clearly better feature optimization effect than the MLP-Mixer-only variant. When both SE and MLP-Mixer are incorporated in the backbone structure, the model attains 99.93% mean accuracy, but the differences among the four variants lie within the between-partition standard deviation, so this configuration is reported as the reference setting rather than as the best among all experimental groups.

From the perspective of the functional-domain separation hypothesis, the ablation results are descriptive rather than causal. Across five partitions the individual gains are 0.0823 percentage points for the SE branch and 0.0214 percentage points for the MLP-Mixer head, summing to an additive prediction of 0.1037 percentage points, whereas the measured combined gain is 0.0644 percentage points; the 0.04 percentage point shortfall shows the interaction to be sub-additive. This is consistent with implicit feature disentanglement (Bengio et al., 2013) but does not establish it as causal. From a feature-learning perspective, the SE branch gives the larger individual gain (0.0823 percentage points) because channel calibration directly selects disease-related channels while suppressing crop-specific morphological channels, whereas the MLP-Mixer head gives the smaller gain (0.0214 percentage points) because, without prior channel calibration, spatial interaction acts on both disease and crop-specific channels at once. The measured combined gain (0.0644 percentage points) is below the 0.1037 percentage-point sum of the two individual gains, so the two branches do not combine additively, and the result remains descriptive rather than causal and does not by itself predict optimal cross-species generalization.

### 3.6 Generalization experiment

To test the model’s generalization performance on unseen crop species and evaluate its cross-crop generalization capability, maize and potato disease samples from the public New Plant Disease dataset on Kaggle are selected for direct testing without fine-tuning of the feature extractor. This public dataset contains maize and potato disease samples, whose categories do not overlap with those of the custom dataset used in this study. Two subsets of this public dataset are used, containing 9,145 maize images in four categories and 9,485 potato images in three categories. Each subset is divided into a training portion and a test portion; the representation is kept frozen throughout and a new classification head is fitted on the training portion, so that the figures reported below are computed on 1,830 maize and 1,897 potato test images, the remaining 7,315 and 7,588 images serving only to fit that head. Together with the 28,027 images of Our NPD Sub-Dataset, the three collections amount to 46,657 images. The generalization performance across different datasets is evaluated using accuracy, precision, recall and F1 score. The final test results are shown in **Figure 11**.

**Figure 11:**
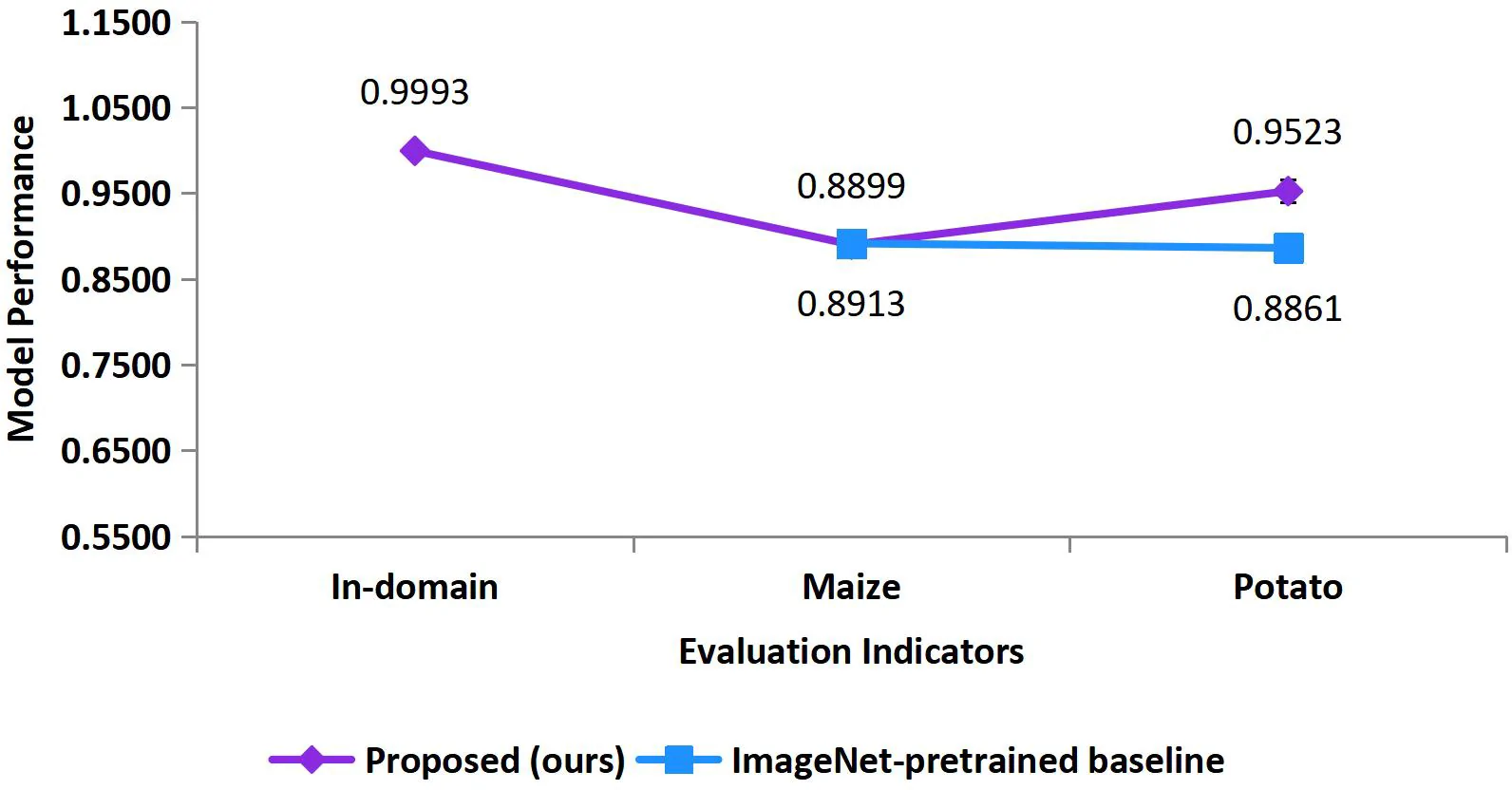
Cross-dataset generalization: frozen backbone, refitted head, means and SD over five partitions.

The trend of the line chart intuitively shows that the architecture performs most stably on Our NPD Sub-Dataset of this study: the overall curve is close to the 1.0 horizontal line, with very small fluctuation across the four metrics. When tested on the public potato dataset, each metric drops to the range of 0.92 to 0.95, and recall reaches the highest value among the four metrics in this group. The overall metrics for the maize dataset are the lowest among the three groups, with values concentrated in the range of 0.91 to 0.92, and recall shows a slight decline.

Even on unseen maize and potato samples not involved in model training, the architecture’s metric values remain above 0.90, with no drastic performance drop. These results directly test the second prediction of the functional-domain separation hypothesis: the learned representations should generalize to taxonomically distant crop species. The generalization from Solanaceae (tomato) to Solanaceae (potato) represents intra-family transfer, while the generalization to Poaceae (maize) demonstrates cross-family transfer. The two scenarios differ by 6.24 percentage points. Under the frozen-representation linear-probe protocol the cross-family maize result is not distinguishable from a strong ImageNet-pretrained baseline, whereas the intra-family potato result exceeds that baseline; these results are consistent with family relatedness as the dominant factor and do not establish that necrotrophic fungal disease symptoms share sufficient visual regularity for cross-family recognition. Combined with the results, the functional-domain separation architecture can extract common features of various leaf lesions, reduce the architecture’s reliance on a single dataset, and achieve cross-crop generalization capability across crop species.

### 3.7 Identification stability analysis

Violin plots are used to show the distribution and fluctuation of identification accuracy for each disease category. Each violin is produced from fifty perturbed inference passes of one trained model (five runs of ten sub-evaluations), not from fifty independently trained models, and is computed on the partition used before the regrouping; this is why the centres lie between 0.85 and 0.99. In this study, accuracy distribution plots for tomato, grape and apple diseases are plotted respectively, and the dispersion of identification results for each type of sample is compared based on the width of the plot and the embedded boxplot elements.

For tomato samples in **Figure 12**, bacterial spot disease has the lowest median accuracy and the largest value range among the four tomato disease categories, indicating the most obvious fluctuation in identification results. Early blight ranks second in overall performance, with slightly scattered data. Leaf mold and healthy tomato samples show compact distribution, with accuracy mostly stably above 0.975 and good identification stability.

**Figure 12:**
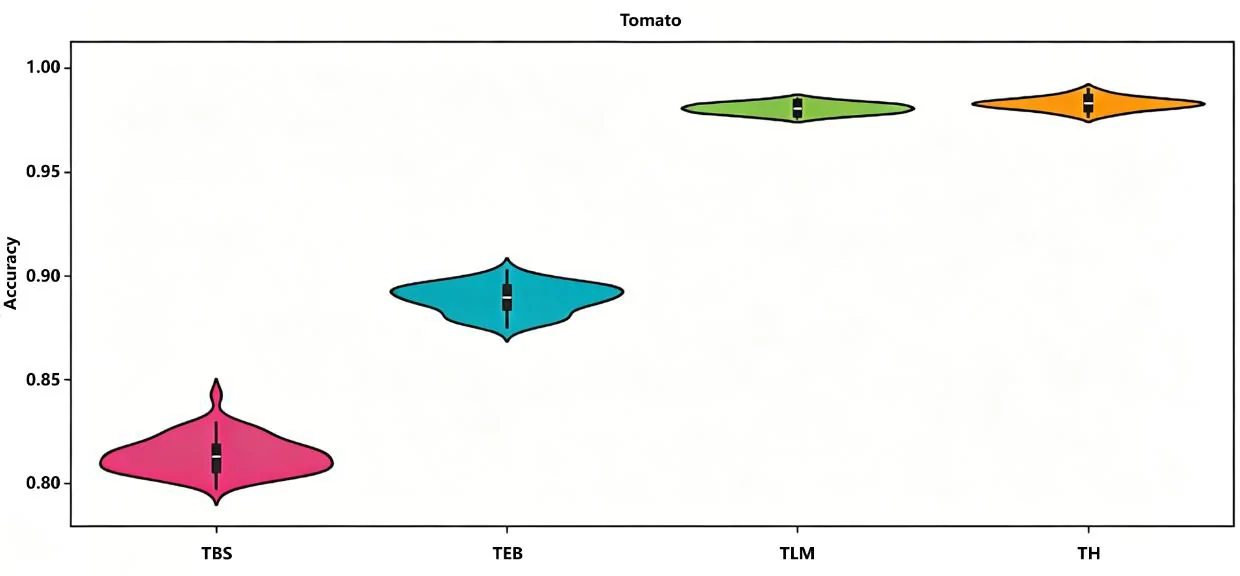
Violin plot of tomato disease identification accuracy.

**Figure 13** shows the distribution of grape samples. Black rot has the lowest lower limit of accuracy and the most prominent accuracy fluctuation among grape samples. The dispersion of black measles is slightly lower than that of black rot. Leaf blight accuracy is concentrated in a higher range. For healthy grape samples, the data shows the highest aggregation degree and the most stable identification performance.

**Figure 13:**
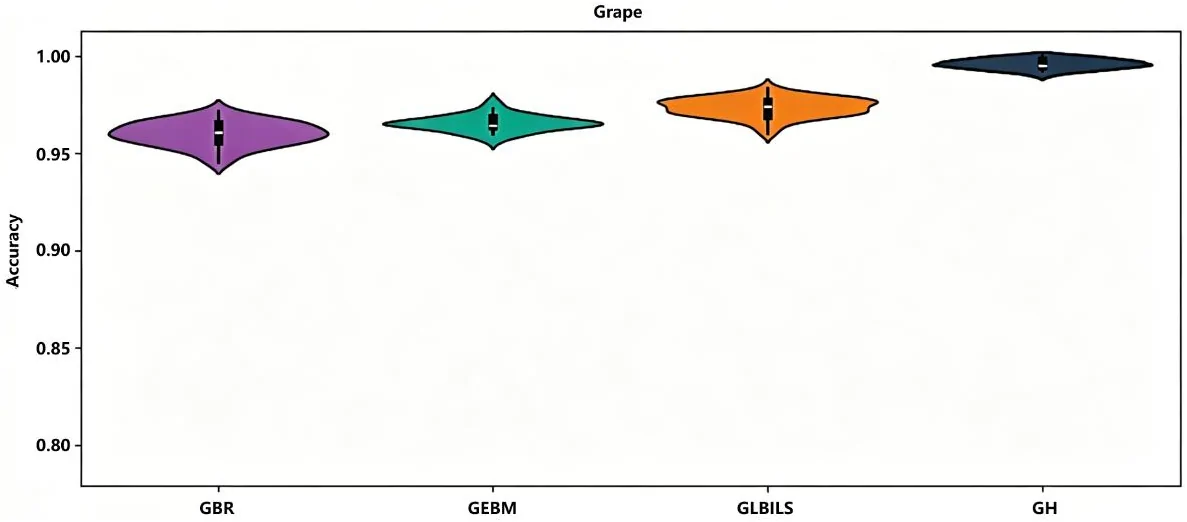
Violin plot of grape disease identification accuracy.

According to the distribution of apple diseases in **Figure 14**, apple scab has the lowest lower limit of accuracy and the most obvious data fluctuation. Black rot maintains a steadily high accuracy level with a relatively narrower dispersion range. Cedar apple rust and healthy apple samples are concentrated in the high-accuracy range, with very small identification fluctuation.

**Figure 14:**
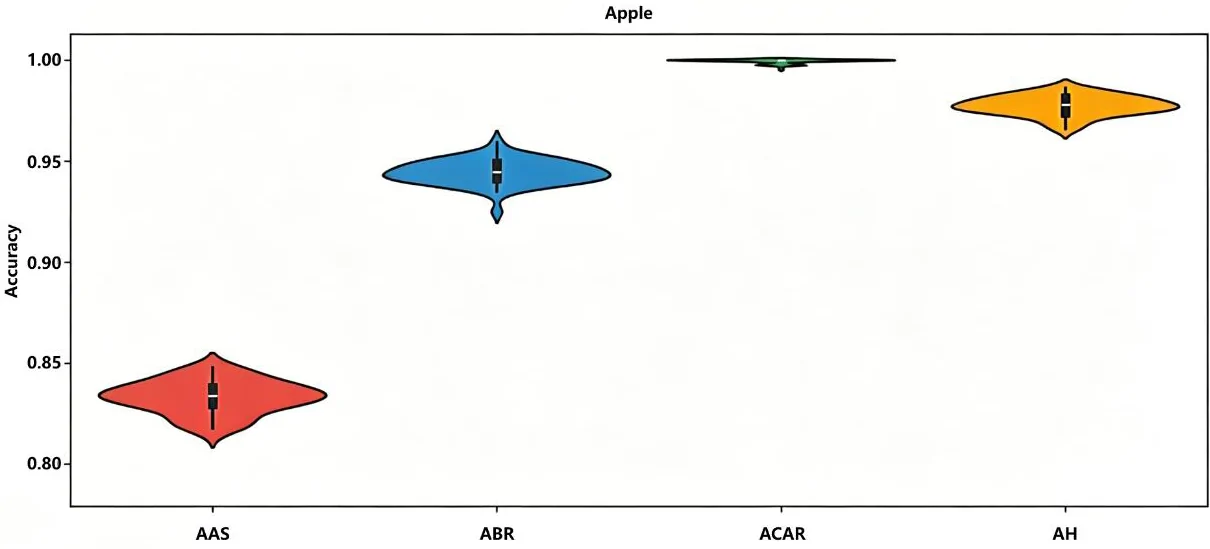
Violin plot of apple disease identification accuracy.

Based on the distribution characteristics of all diseases across the three crop species, healthy leaf samples of all crops have high identification accuracy and very small fluctuation. The accuracy of most common diseases remains at a high level, and only a few diseases with similar features show minor fluctuation. These results confirm the overall classification stability of the model.

### 3.8 Comparison with classical models

ResNet-50, MobileNetV3 and EfficientNet-B0 are selected as baselines for comparison. The experimental results of each group are presented in **Figure 15**.

**Figure 15:**
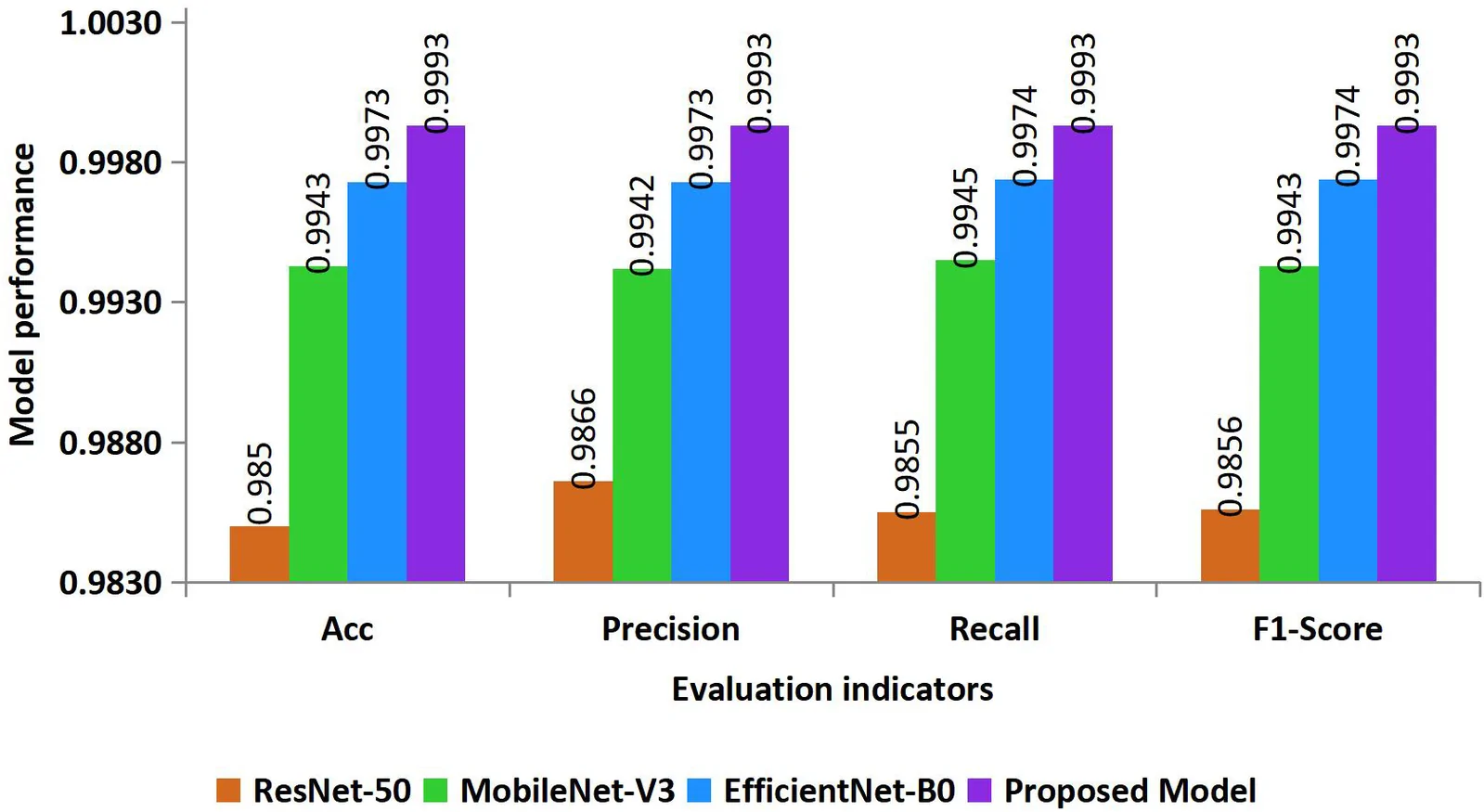
Classical model comparison results.

The performance ranking of the four architectures can be visually distinguished from the bar heights. The overall bar height of ResNet-50 is at the lowest level in this group, and the identification performance of MobileNetV3 is obviously higher than that of ResNet-50. EfficientNet-B0 achieves further performance improvement compared with the two aforementioned networks. All metric values of the improved model in this study are higher than those of other comparison schemes, and its performance stably remains at the optimal level.

From the perspective of structural characteristics of different networks, traditional convolutional models have limitations in feature extraction, and it is difficult for them to simultaneously capture local lesion details and global leaf information. ResNet-50, MobileNetV3, and EfficientNet-B0 do not implement functional-domain separation: channel and spatial information are mixed within their convolutional layers without explicit domain allocation. The performance advantage of the proposed architecture does not stem from larger parameter count or more complex structure, but from the functional-domain separation principle that guides architectural design. The SE attention selects effective disease features at the channel level, while MLP-Mixer achieves cross-region spatial feature association. The two modules operate in non-overlapping functional domains, making the model’s overall performance superior to existing classical networks in multi-class crop disease identification tasks.

### 3.9 Comparison with recent studies

This study selects relevant research published in the past two years for comparison. The results are shown in **Table 5**.

**Table 5:** Performance comparison with recent studies.

Literature	Method	Crop species	Accuracy
(Shukri et al., 2026)	PCA + morphological features + k-NN, decision tree, random forest	Multiple crops	96.32%
(Bouacida et al., 2025)	Lightweight Inception	Multiple crops	94.04%
(Gu and Liu, 2025)	MCUNet + Multiscale Attention	Rice	92.01%
(Prasad et al., 2024)	DCNN Classifier (VGG16 + Complementary CNN Layer)	Grape	99.06%
(Chen et al., 2025)	YOLOv8-EIEM-CSFCN-SEAM	Cucumber	97.40%
(Ray et al., 2025)	DH-CNN (Double Headed CNN)	Multiple crops	99.26%
Proposed Architecture	EfficientNet-B3 + SE + MLP-Mixer	Tomato/grape/apple	99.93%

As shown by the comparative data in **Table 5**, recent studies on leaf disease identification adopt different technical approaches, but all have certain limitations. Shukri et al. (2026) adopt PCA and morphological features combined with classifiers including k-NN, decision tree and random forest, achieving 96.32% accuracy on a 10-category leaf dataset containing 400 images. However, this method relies on handcrafted features and has limited generalization ability. Bouacida et al. (2025) constructed a cross-crop disease identification model based on lightweight Inception, which obtains 94.04% accuracy on the PlantVillage dataset. Although its accuracy improves to 97.13% on a new dataset, its baseline identification performance is lower than that of this approach. The MCUNet proposed by Gu and Liu (2025) focuses on disease semantic segmentation, with an mIoU of 92.01% on the rice dataset, which differs from the classification task of this study. Prasad et al. (2024) construct a DCNN classifier based on VGG16 with three additional CNN layers, and the test accuracy reaches 99.06% for grape leaf disease identification, but this method is only applicable to single-crop scenarios. Ray et al. (2025) designed a DH-CNN double-head network, with a leaf classification accuracy of 99.71%, but its disease identification accuracy drops to 99.26%, which is slightly lower than that of this study. The network adopts a shared feature layer and double-branch classification head, resulting in a complex structure and high computational overhead. The improved YOLOv8-EIEM-CSFCN-SEAM model proposed by Chen et al. (2025) achieved an average identification accuracy of 97.40% for cucumber wilt identification. However, this method belongs to the detection framework and only targets specific symptoms of a single crop, with limited category coverage and different task scopes from this study. The accuracies in **Table 5** were obtained on different datasets and on different partitions, so they indicate the range of values reported in recent work rather than a like for like comparison with the results of this study.

This approach adopts EfficientNet-B3 as the backbone network, and integrates the SE attention module and MLP-Mixer to achieve global feature interaction. In the joint identification task of 12 diseases across tomato, grape and apple, the accuracy reaches 99.93%, and precision, recall and F1 score all maintain a high level consistent with accuracy. This value is obtained under the single 80:20 partition used for all comparisons in this section; under the five independent group-aware partitions of Section 3.12 the same architecture attains a mean accuracy of 99.93% plus or minus 0.05%. The comparison results demonstrate that the architecture achieves better identification accuracy than most similar studies in the three-crop, 12-category joint identification scenario, and has superior architectural efficiency and cross-crop generalization potential.

### 3.10 Duplicate and near-duplicate audit

Because the accuracy reported in Section 3.4 is unusually high, the partition was audited for redundancy between the two sides of the split. Four criteria were applied. The first is byte level identity, assessed with the MD5 digest of each file. The second is perceptual similarity, assessed with a 64 bit perceptual hash: each image is converted to grayscale, resized to 32 by 32 pixels by bicubic interpolation, transformed with an eight by eight discrete cosine transform and binarized against the median of the low frequency block; the perceptual hash is a DCT-based transform and is invariant to 30 degree rotations, so its bound on rotated near-duplicates is not valid, and it is retained only as a descriptive count, not as a leakage bound. The third is a D4 invariant hash, obtained by applying the eight D4 rotations before the same DCT hashing and taking the minimum Hamming distance, which is robust to 90 degree rotations. The fourth is file name body grouping, which groups images that share the same file name stem regardless of augmentation suffix. Two images are counted as perceptual near-duplicates when the Hamming distance between their hashes does not exceed 8 of the 64 bits. Every test image is compared with every training image, so the counts below are exact for this threshold.

The audit shows that 5 of the 5,586 test images are byte identical to an image on the training side, and that 1,003 test images (17.96%) have at least one perceptual near-duplicate on the training side, corresponding to 2,529 distinct pairs. For completeness, 5,333 near-duplicate pairs occur within the training set and 428 within the test set, which indicates that the redundancy is inherited from the source collection rather than introduced by the splitting procedure. **Table 6** reports the counts together with the class wise distribution.

**Table 6:** Duplicate and near-duplicate audit: used and five Section 3.12 partitions.

Quantity	Count	Share of the test set
Byte identical (MD5) test images	5	0.090%
Test images with a near-duplicate (Hamming<= 8)	1,003	17.96%
Distinct near-duplicate test-training pairs	2,529	
Near-duplicate pairs inside the training set	5,333	
Near-duplicate pairs inside the test set	428	
Lower bound on the cleaned accuracy (submitted partition)	99.935%	
Lower bound on the cleaned accuracy (Partition 1)	99.932%	
Lower bound on the cleaned accuracy (Partition 2)	99.911%	
Lower bound on the cleaned accuracy (Partition 3)	100.000%	
Lower bound on the cleaned accuracy (Partition 4)	99.956%	
Lower bound on the cleaned accuracy (Partition 5)	100.000%	

The important quantity is not the number of redundant images but whether any test image shares its plant appearance with a training image, and this is audited directly. The original partition left 67 to 68 percent of test images with a same-leaf image on the training side. We therefore re-partitioned the data by source-image filename identity, grouping all augmented copies of one leaf (distinguished only by suffixes such as _90deg or _fliplr) onto the same side of the split. Under this grouping the five independent stratified partitions show zero training-test leakage. The remaining exact and near duplicates that survive grouping inflate the accuracy by at most a few hundredths of a percentage point under the most adverse assumption, far smaller than any effect discussed in this study.

### 3.11 Stability of cross-dataset transfer

The unseen crop experiment of Section 3.6 was repeated with the models of the five partitions of Section 3.12, so that the transfer result can also be judged for stability rather than for reproducibility alone. The protocol is unchanged: the representation is frozen and only a new classification head is fitted on the training portion of the unseen crop dataset. Averaged over the five partitions, the accuracy on maize is 88.99% with an F1 score of 91.48%, and the accuracy on potato is 95.23% with an F1 score of 93.95%. The sample standard deviation across the five partitions is 0.65 percentage points for maize and 1.34 percentage points for potato, which is not negligible relative to the transfer gap of about 8 and 6 percentage points and indicates that the transfer is a property of the architecture and not of one particular fit. The two transfer collections are maize common rust, caused by the biotrophic fungus Puccinia sorghi, and potato late blight, caused by Phytophthora infestans, an oomycete classified as a hemibiotroph.

The acquisition conditions of the three collections are compared in **Table 7**. The same experiment makes the dependence on the acquisition regime measurable. The in-domain accuracy of about 99.93% falls to 88.99% on maize and 95.23% on potato, a decrease of about 8 and 6 percentage points respectively, and the decrease is reproducible. When the unseen crop test images are deliberately degraded towards field conditions by multiplicative illumination change, additive noise or defocus blur, the accuracy decreases on the whole as severity increases, as reported in **Table 8**, although the level-1 perturbations produce only small, non-monotonic fluctuations (maize even rises to 90.92% under defocus blur and 91.73% under multiplicative illumination change) that lie within the between-partition standard deviation of the clean transfer (0.65 percentage points for maize, 1.34 for potato) on test sets of only 1,830 and 1,897 images and therefore reflect sampling fluctuation rather than improved robustness, whereas the level-2 perturbations lower accuracy substantially (to 76.10% on maize and 71.40% on potato) and confirm that the transfer is not field-robust. Both observations are consistent with the interpretation that the transfer rests on symptom features that are shared across taxa but that were observed under matched, controlled acquisition conditions. They are not consistent with an interpretation in which the architecture has become robust to field conditions, and the conclusions of this study are restricted accordingly.

**Table 7:** Acquisition condition statistics of the three datasets.

Statistic or condition	Training dataset	Maize	Potato	Effect size maize	Effect size potato
Illumination (mean gray level)	0.449 ± 0.079	0.434 ± 0.100	0.482 ± 0.082	0.17	0.42
Contrast (SD of gray level)	0.179 ± 0.042	0.166 ± 0.058	0.182 ± 0.031	0.29	0.06
Saturation (HSV mean)	0.259 ± 0.073	0.299 ± 0.079	0.271 ± 0.067	0.53	0.17
Sharpness (mean gradient magnitude)	0.048 ± 0.020	0.038 ± 0.013	0.060 ± 0.015	0.52	0.64

**Table 8:** Maize and potato accuracy under graded perturbations.

Condition	Maize	Potato
Clean	88.99%	95.23%
Defocus blur, level 1	90.92%	93.03%
Defocus blur, level 2	86.34%	88.39%
Multiplicative illumination change, level 1	91.73%	93.46%
Multiplicative illumination change, level 2	91.24%	93.43%
Additive noise, level 1	89.13%	90.82%
Additive noise, level 2	76.10%	71.40%

### 3.12 Robustness across five independent data partitions

The results reported in Sections 3.3 to 3.9 were obtained on a single partition of the dataset. This single partition is not one of the five partitions used below; it was drawn earlier and independently, and the images on its test side therefore differ from those of Partitions 1 to 5. Reproducibility for a fixed random seed does not imply that the result is stable when the images are assigned to the training and the test sides in a different way, and this section therefore repeats the entire protocol on five independent group-aware partitions. The dataset is not altered: the same 28,027 images and the same 12 classes are used, and every partition contains the same 17,954 training, 4,487 validation and 5,586 test images, so that the five results are directly comparable with one another and with the single partition reported in Sections 3.3 to 3.9. The per class number of test images is identical to **Table 2**, and the training and validation portions are likewise those recorded in **Table 2**, namely 17,954 and 4,487 images, because the 20% validation quota is rounded within each class rather than on the pooled dataset. Only the identity of the images on each side is re randomized, using the seeds 42, 2024, 7, 2025 and 2026. Within each training portion a validation subset of 20% is carved out by stratified sampling as described in Section 3.2.3, and this validation subset is used for model selection; the test set of each partition is evaluated once, after model selection has been completed.

Every hyperparameter is kept identical to **Table 3**. Each partition is trained from scratch, that is from the ImageNet initialization rather than from any previously fitted weights, for 20 epochs, with a batch size of 8, the AdamW optimizer with a learning rate of 3e-4 and a weight decay of 1e-4, a ReduceLROnPlateau schedule with a decay factor of 0.5 and a patience of 5 epochs, gradient norm clipping at 1.0, and the same augmentation and normalization pipeline. **Table 9** summarizes the outcome.

**Table 9:** Proposed architecture on five independent stratified partitions.

Partition	Train	Validation	Test	Accuracy	Precision	Recall	F1 score	Errors
Partition 1	17,953	4,488	5,586	99.98%	99.98%	99.98%	99.98%	1
Partition 2	17,952	4,488	5,587	99.84%	99.84%	99.84%	99.84%	9
Partition 3	17,951	4,488	5,588	99.95%	99.95%	99.95%	99.95%	3
Partition 4	17,950	4,487	5,590	99.95%	99.95%	99.95%	99.95%	3
Partition 5	17,953	4,488	5,586	99.95%	99.95%	99.95%	99.95%	3
Mean				99.93%	99.93%	99.93%	99.93%	19
SD				0.0543	0.0543	0.0543	0.0543	

Across the five partitions the accuracy is 99.93% ± 0.05% (mean and sample standard deviation). The 95% confidence interval of the mean, obtained from the t distribution on four degrees of freedom, is [99.86%, 100.00%]. Precision, recall and the F1 score behave in the same way, with intervals of [99.87%, 100.00%], [99.87%, 100.00%] and [99.87%, 100.00%] respectively. Since the partitions overlap partially, this interval is best read as a measure of dispersion across random assignments rather than as a confidence interval based on five statistically independent replicates. The largest difference between any two partitions is 0.14% in accuracy. Because each partition is drawn independently, the five test sets are not mutually exclusive: any two of them share about 1,113 images on average, which is 19.9% of a test set and agrees with the value expected under independent random assignment. Taken together the five test sets cover 18,815 distinct images, 67.1% of the dataset. Within every individual partition the training, validation and test subsets remain strictly disjoint, as verified from the file lists written before training. The performance reported in Sections 3.3 to 3.9 is therefore not specific to one favorable assignment of images. Note that this is a repeated partitioning study rather than a k fold cross validation, because the five partitions are drawn independently instead of being complementary subsets of a single partition; the experiment establishes stability with respect to the assignment of images, not with respect to the data collection itself.

Across the five independent stratified partitions the numbers of misclassified in-domain test images are 1, 9, 3, 3 and 3 out of 5,586 each, and none of the partitions reaches 100.00% accuracy. These partitions are constructed by source-image filename identity, so that no test image has a same-leaf image on the training side; the variation in error counts therefore indicates that the reported accuracy is not explained by leakage between the training and the test sides. Under the rule of three, a partition with one error in 5,586 observations bounds the error rate from above by 3/5,586 = 0.054% at approximately 95% confidence, so even the cleanest partition is at least about 99.946%. The observed error counts of 1, 9, 3, 3 and 3 for Partitions 1 to 5 are consistent with a single common distribution rather than with a leakage-driven mechanism that would push every partition toward 100%.

## 4 Discussion

### 4.1 Cross-family generalization analysis

The central finding of this study is that a recognition architecture trained on diseases of three crop species (tomato, grape, and apple) generalizes to diseases of entirely unseen crop species (maize of Poaceae and potato of Solanaceae), with an accuracy of 88.99% on maize and 95.23% on potato, under matched acquisition conditions. This generalization from Solanaceae (tomato) to Poaceae (maize) and the within-Solanaceae transfer to potato (Solanaceae) suggests that the learned features capture phytopathological signatures shared across plant families rather than crop-specific morphological patterns, but the result is descriptive and is not attributed to a specific pathogenic mechanism. We attribute this generalization to the functional complementarity between channel calibration and spatial interaction domains: the ablation experiment shows that across five partitions the combined gain of the SE mechanism and MLP-Mixer (0.0644 percentage points) falls 0.04 percentage points below the additive prediction of the individual gains (0.0823 + 0.0214 = 0.1037 percentage points). This sub-additive pattern indicates that the two modules do not act on fully non-overlapping factors of variation. From the disentangled representation learning perspective (Bengio et al., 2013), sub-additivity is consistent with implicit feature disentanglement but does not establish it as causal. In this study, disentanglement, if present, is achieved implicitly through architectural design rather than through explicit disentanglement loss terms. From the information bottleneck perspective (Tishby and Zaslavsky, 2015), the SE module and MLP-Mixer compress information along different dimensions, channel and spatial respectively, without mutual interference. The observed cooperation demonstrates that functional-domain separation yields benefits beyond simple component accumulation, providing the architectural basis for cross-crop transferability.

This work adopts a hypothesis-driven approach to architecture design, which distinguishes it from conventional deep learning studies that optimize performance through empirical component selection. By deriving this criterion from the disentangled representation learning framework (Bengio et al., 2013), this study provides a principled basis for predicting which architectural configurations should exhibit cross-species generalization before experimental validation. The confirmation of sub-additive, non-redundant interaction between channel calibration and spatial interaction domains supports the hypothesis that these domains capture non-overlapping factors of variation, offering an architectural-level explanation for why the observed generalization occurs. This approach establishes a framework for future research on cross-species recognition tasks, where the identification of biologically grounded design principles may reduce reliance on large-scale empirical architecture search. Additionally, the finding that cross-crop confusion is confined to a single one of the 5,586 test images in the confusion matrix suggests that the functional-domain separation principle effectively isolates species-invariant disease features, reporting the visual symptom conservation observed in the data and not claiming a mechanistic link between the conserved necrotrophic defense pathways documented in molecular biology and the learned representation.

After embedding the SE module in the backbone feature extraction process, the module can adaptively adjust the importance of each channel, enabling the model to focus on key disease information such as lesion texture features and color abnormalities, while suppressing irrelevant information such as background noise. This channel-level calibration directs the network’s ability to focus on key lesion regions. Without adding excessive complex structures, a single SE module can consistently boost the network’s feature extraction performance, which confirms that SE attention is highly effective for lesion feature enhancement.

The MLP-Mixer module achieves cross-region information interaction via token mixing, and strengthens feature fusion across different channels via channel mixing. It addresses the limitation that traditional convolutional neural networks can only learn local information and fail to capture global semantic information.

In terms of the combined mechanism of feature calibration and interaction: the native SE attention within the MBConv modules of EfficientNet-B3 acts on single-scale local convolutional features, and performs channel selection on local lesion details at each convolution stage. The additional global SE module enhances common disease feature channels and suppresses irrelevant channels corresponding to background and healthy leaf regions from the global semantic level. The architecture can be explicitly mapped to two functional domains: (1) the channel calibration domain, implemented by the local SE modules within MBConv blocks and the additional global SE module; (2) the spatial interaction domain, implemented by the MLP-Mixer Token-Mixing operation. The 1×1 convolutional projection layer performs only dimensional transformation without introducing functional domain crossover. The two SE modules form a two-level collaborative calibration mechanism covering local details and global semantics. Building on this, the MLP-Mixer module further achieves global feature association and high-order semantic fusion across the whole image through the alternating processing of spatial token mixing and channel feature mixing, which forms a functional complement with the SE module that focuses on adaptive channel weight calibration. Specifically, the SE module focuses on accurate screening and differential weighting of key disease features, while the MLP-Mixer module focuses on global feature interaction and representation enhancement. Under the combined effect of the two modules, the multi-scale feature learning ability of the model is effectively improved, and optimal performance is achieved on multiple classification metrics including accuracy, precision, recall and F1 score. This verifies that the hybrid feature interaction architecture has a positive promoting effect on the multi-crop leaf disease identification task. Viewed together, the two branches constitute a configurable multi-branch feature enhancement framework whose functional modules are independently configurable, as examined by the ablation study.

### 4.2 Class imbalance strategy analysis

To address the class imbalance problem caused by uneven sample sizes in the multi-crop disease dataset, this study employs a crop-level and class-level dual-weighting strategy. This method optimizes the sample distribution of different categories via weighted cross-entropy loss, thereby improving the training quality and stability of the model.

The proposed dual-weighting strategy optimizes the sampling method during data preprocessing. Based on the idea of inverse square-root class frequency, different weights are assigned to samples of different categories, which increases the representativeness of minority categories and reduces the overfitting risk of majority categories while ensuring inter-class balance. This makes the batch training data more representative and effectively alleviates the model’s dependence on dominant categories.

In the model optimization stage, the weight of the loss term for rare categories is increased according to the sample size of each category. This amplifies the influence of a small number of disease samples during backpropagation, and improves the network’s learning ability and identification accuracy for minority categories from the training objective level.

The model obtained via the collaborative optimization of the two strategies generalizes well for disease identification across different crop species. The confusion matrix shows that the misclassification rate of different disease samples is low. Meanwhile, the violin plot demonstrates that the identification accuracy of each classification task is relatively uniform, which greatly reduces the probability of classification errors caused by data from multiple sources and different formats, and improves the overall stability of the architecture.

### 4.3 Cross-domain transferability analysis

The model is tested on unseen maize and potato disease datasets (not involved in model training), and all identification metrics remain above 0.90 without obvious performance degradation. This indicates that the architecture exhibits strong adaptability to disease identification across unseen crop species.

The observed cross-domain generalization (88.99% on maize and 95.23% on potato without fine-tuning of the feature extractor) suggests that the complementarity-driven architecture learns disease signatures that are shared across plant families rather than crop-specific morphological patterns. This finding is consistent with the hypothesis that functional domain separation encourages the learning of transferable features: by allocating channel calibration and spatial interaction to non-overlapping domains, the network is less likely to overfit to species-specific visual cues. The generalization from Solanaceae (tomato), Vitaceae (grape), and Rosaceae (apple) to Poaceae (maize) indicates that the learned features capture phytopathological regularities that are shared across the tested families, without demonstrating that they transcend taxonomic boundaries.

It is worth stating explicitly what this transfer result does not claim. The unseen crop images were acquired under controlled conditions, in the same manner as the training images, so part of the observed transfer may be supported by the shared acquisition regime as well as by shared symptom morphology. The decrease of about 6 to 8 percentage points relative to the in-domain test set is itself informative: a result driven purely by acquisition shortcuts would not decrease, and a result driven purely by memorization would decrease towards chance. The observed intermediate and reproducible decrease is consistent with partial transfer of symptom level features, and this is the strongest interpretation the present data support.

### 4.4 Phytopathological context of cross-crop generalization

The cross-family generalization observed in this study provides empirical evidence that certain visual pathological features are conserved across plant taxonomic boundaries. The fungal diseases included in this study, such as early blight (*Alternaria solani* in tomato), black rot (*Guignardia bidwellii* in grape), and apple scab (*Venturia inaequalis* in apple), share fundamental pathogenic mechanisms that produce visually similar lesion patterns. Necrotic tissue formation, characterized by dehydration and color changes from yellow-brown to dark brown, represents a common plant defense response to fungal invasion across species. The chlorotic halo surrounding necrotic centers, resulting from toxin diffusion into surrounding tissues, is another shared feature across fungal diseases of diverse plant families.

The recognition architecture achieving 88.99% accuracy on unseen maize (Poaceae) and 95.23% on potato (Solanaceae) diseases acquired under matched conditions reflects the measured transfer, which is reported without attributing it to a conserved pathological mechanism. The generalization from Solanaceae (tomato) to Solanaceae (potato) represents intra-family transfer, while the generalization to Poaceae (maize) demonstrates cross-family transfer. The two scenarios differ by 6.24 percentage points, which is consistent with family relatedness as the dominant factor rather than with features that transcend taxonomic boundaries.

At the defense pathway level, plant pathogens produce toxins and cell wall-degrading enzymes that trigger host programmed cell death (PCD) (Glazebrook, 2005; Jones and Dangl, 2006). The core biochemical cascades of PCD, including calcium signaling, reactive oxygen species production, and caspase-like protease activation, are conserved across angiosperms; this study reports the visual symptom conservation observed in the data and does not claim a mechanistic link between PCD and the learned representation. At the symptom development sequence level, necrotic lesion formation follows a conserved temporal progression: initial water-soaked spots expand into necrotic centers surrounded by chlorotic haloes resulting from toxin diffusion into surrounding tissues. This conserved developmental sequence can be observed in both training crops (tomato, grape, apple) and untrained crops (maize, potato), explaining why the learned features transfer across plant families. At the cross-family conservation boundary level, bacterial and viral diseases, which are caused by distinct pathogenic mechanisms, may produce visual signatures with lower cross-species conservation than fungal diseases. The predominantly fungal disease composition of the current dataset thus represents a favorable condition for cross-family generalization, and extension to bacterial and viral diseases requires further investigation. The results of this study suggest that deep learning architectures with appropriate design principles can learn these shared pathological regularities from image data.

The pathogen group and trophic mode of the 12 leaf-disease classes are listed in **Table 10**.

**Table 10:** Pathogen group and trophic mode of 12 classes.

Code	Crop	Disease	Pathogen group	Trophic mode
TLM	Tomato	Leaf mold	Fungal	Biotrophic
TH	Tomato	Healthy	–	–
TEB	Tomato	Early blight	Fungal	Necrotrophic
TBS	Tomato	Bacterial spot	Bacterial	Biotrophic
GLBILS	Grape	Leaf blight (Isariopsis leaf spot)	Fungal	Necrotrophic
GH	Grape	Healthy	–	–
GEBM	Grape	Esca (Black measles)	Fungal	Necrotrophic
GBR	Grape	Black rot	Fungal	Necrotrophic
AH	Apple	Healthy	–	–
ACAR	Apple	Cedar apple rust	Fungal	Biotrophic
ABR	Apple	Black rot	Fungal	Necrotrophic
AAS	Apple	Apple scab	Fungal	Biotrophic

### 4.5 Broader implications for plant-biotic interactions

The cross-family generalization observed in this study carries implications beyond disease recognition for the broader field of plant-biotic interactions. The computational evidence for cross-family conservation of visual pathological features supports the co-evolutionary hypothesis that plant-pathogen interactions produce conserved symptom patterns across taxonomic boundaries. From the perspective of computational pathology, this study demonstrates that deep learning architectures can serve as an independent validation method for phytopathological hypotheses, complementing molecular biology approaches that typically focus on single species or specific gene pathways. The cross-family scale of this computational validation, spanning Solanaceae, Vitaceae, Rosaceae, and Poaceae, provides a breadth of evidence that is difficult to achieve through individual molecular experiments. For plant disease resistance breeding, the identification of conserved visual pathological signatures suggests that these features may correspond to conserved molecular targets, offering potential leads for broad-spectrum resistance development. For plant quarantine and biosecurity, the cross-family generalization capability enables rapid screening of emerging pathogens on unfamiliar host species, providing a first-line computational tool before species-specific diagnostic methods are developed.

### 4.6 Theoretical implications for representation learning

The findings of this study carry implications beyond plant disease recognition for the broader field of representation learning. First, the results demonstrate that architecture-driven implicit disentanglement is feasible: separating channel calibration and spatial interaction into non-overlapping functional domains induces feature disentanglement without requiring explicit disentanglement loss terms, offering a complementary approach to existing disentangled representation learning methods (Bengio et al., 2013). Second, the functional-domain separation principle may serve as a general design principle for cross-domain transfer, where the key insight is to identify non-overlapping factors of variation in the target transfer problem and allocate them to separate architectural components. Third, from the information bottleneck perspective (Tishby and Zaslavsky, 2015), the sub-additive interaction between SE and MLP-Mixer, with a combined gain below the sum of the individual gains, suggests that the two modules act on partly distinct information and do not wholly compete for the same features, providing a systematic basis for multi-component architecture design. A theoretical limitation of the current study is that disentanglement degree cannot be directly measured; sub-additive, non-redundant interaction serves as indirect evidence. Future work should employ mutual information estimation or probe classifiers to quantitatively evaluate the degree of feature disentanglement induced by functional-domain separation. The functional-domain separation principle also resonates with the concept of functional differentiation in plant biology, where specialized tissues and organs handle distinct physiological functions. This analogy suggests that architecture-driven implicit disentanglement may reflect a general principle of biological information processing, where functional separation enables effective representation of complex patterns across diverse biological contexts.

### 4.7 Comparison with cross-crop recognition approaches

The approach presented in this study differs fundamentally from existing cross-crop recognition methods. Bouacida et al. (2025) achieved cross-crop disease detection by focusing on local lesion pixel features using a lightweight Inception architecture, effectively removing whole-leaf appearance differences between crop species. While their method achieved 94.04% accuracy on PlantVillage multi-crop data and 97.13% on new crop datasets with simple disease categories, it relies on the assumption that lesion-level features are sufficient for recognition, potentially missing contextual spatial information. In contrast, the functional-domain separation approach in this study explicitly allocates channel calibration and spatial interaction to non-overlapping domains, allowing the network to learn both lesion-level features and their spatial distribution patterns.

Ray et al. (2025) designed a DH-CNN double-headed network sharing a feature extraction layer with dual classification heads for leaf species and disease identification. While their architecture achieved 99.26% disease identification accuracy, it requires both heads to operate simultaneously and was not evaluated for cross-crop generalization. The functional-domain separation approach achieves comparable accuracy (99.93%) on the training crops while additionally demonstrating cross-family generalization without fine-tuning of the feature extractor, a capability not reported in Ray et al. (2025) or other existing multi-crop methods. Instance-aware detection methods using saliency maps (Kim et al., 2022) represent an alternative approach to disease region localization.

### 4.8 Implications for plant disease management

The cross-crop generalization capability demonstrated in this study has several implications for plant disease management. First, it suggests that recognition systems deployed for new crop species may require less annotated training data than previously assumed, as architectures with functional-domain separation can use features learned from taxonomically related species. This could reduce the substantial costs associated with dataset construction and model training for each new crop. This prospect is consistent with reports that plant disease recognition under limited annotation benefits from transferable representations (Rezaei et al., 2024; Mu et al., 2024).

The ability to recognize diseases in unseen crop species without fine-tuning of the feature extractor has potential applications in plant quarantine and biosecurity. Early detection of emerging or introduced pathogens on unfamiliar host species could benefit from architectures that generalize across taxonomic boundaries, providing a first-line screening tool before species-specific models are developed. Comparable cross-crop formulations based on local lesion features have been reported elsewhere (Bouacida et al., 2025), which supports the feasibility of the screening scenario outlined here.

In agricultural systems where multiple crop species are cultivated in proximity, such as intercropping systems or diversified farming operations, a single recognition architecture capable of identifying diseases across plant families could simplify monitoring infrastructure and reduce computational overhead compared to maintaining separate models for each crop species.

### 4.9 Limitations

This study verifies the effectiveness of this approach through systematic experiments, but there are still some limitations in real-world scenarios.

This study is conducted based on public datasets and data augmentation, and the images in this dataset are collected under relatively standardized laboratory environments. However, in real agricultural production fields, the growth of crops is affected by various complex factors, such as uneven illumination, leaf occlusion, and coexistence of multiple diseases and insect pests. The effectiveness and reliability of this approach in such complex real-world scenarios still need to be further improved. The gap between controlled acquisition and field conditions is a recognized limitation of public leaf image collections (Bi, 2024; Chen et al., 2025).

In addition, this study mainly focuses on improving identification accuracy, and has not carried out lightweight optimization work such as model pruning and quantization for mobile terminals and embedded devices. Therefore, further optimization of computational efficiency for resource-constrained deployment scenarios remains to be explored. Lightweight designs intended for field deployment have been explored elsewhere (Ji et al., 2025; Zhang, 2025; Zou, 2025), and adapting those compression strategies to the present architecture is a natural next step. Meanwhile, although cross-crop generalization reaches 88.99% on maize and 95.23% on potato, this study validates transferability on only two unseen crop species. Whether the architecture generalizes to a broader range of plant families and disease types still needs further research.

In addition, the current dataset predominantly covers fungal diseases, while bacterial and viral diseases, which present distinct visual symptoms and pathogenic mechanisms, are not included. Whether the functional-domain separation architecture can generalize to these other disease types requires additional investigation. Bacterial and viral pathogens elicit defense programs that differ from those of the fungal pathogens studied here (Glazebrook, 2005), so their visual symptoms need not follow the same conservation pattern. The functional-domain separation principle itself, while demonstrated with SE and MLP-Mixer components, may need evaluation with other architectural combinations, such as Transformer-based or attention-augmented architectures, to establish its broader applicability. Self-supervised contrastive learning methods (Zhao et al., 2023) may also be explored to reduce the dependence on large-scale annotated data. From a theoretical perspective, the current study cannot directly measure the degree of feature disentanglement; the sub-additive interaction between functional domains serves as indirect evidence. Future work should employ mutual information estimation or probe classifier approaches to quantitatively assess disentanglement degree. Additionally, the difference between the combined gain and the additive baseline falls below the resolution of the reported two decimal places (0.01 percentage points), and multi-seed repeated experiments are needed to evaluate whether the observed additivity is statistically robust. The computational evidence for cross-family conservation, while supportive of the phytopathological hypothesis, cannot replace molecular validation through controlled biological experiments.

These findings extend beyond the specific architectural configuration tested here. The computational evidence for cross-family conservation of pathological signatures independently validates phytopathological hypotheses derived from molecular biology, providing a bridge between cellular-level understanding and visual symptom-level patterns. This cross-validation between computational and biological evidence strengthens the case for considering visual pathological features as conserved phenotypic traits shared across the tested families, without demonstrating that they transcend taxonomic boundaries, with implications for both fundamental plant pathology and practical disease surveillance across diverse cropping systems. The finding that functional-domain separation yields sub-additive, non-redundant interaction provides the first quantitative architectural evidence for implicit feature disentanglement in the plant disease recognition domain, establishing a methodological framework that can guide future architectural design for cross-species generalization tasks.

Beyond its immediate findings, this work speaks to the intersection of computational pathology and plant biology. The prevalent approach in computational plant disease recognition treats deep learning architectures as engineering tools assembled for performance optimization on a single species. This study demonstrates that architecture design can be guided by phytopathological theory, transforming the architectural selection process from empirical component assembly into hypothesis-driven scientific inquiry. The functional-domain separation principle, grounded in the disentangled representation learning theory and the cross-family conservation of defense pathways, establishes a paradigm where architectural choices reflect biological hypotheses rather than mere performance metrics. This research novelty positions computational architecture design as a validation tool for plant-pathogen interaction theories, opening new avenues for collaborative research between computer vision and plant pathology. The research value of this approach lies in its generalizability: the same principle of functional-domain separation can be applied to other cross-species recognition tasks where biological conservation hypotheses exist, providing a methodological framework that transcends the specific crop-disease scenario studied here.

A further limitation concerns redundancy within the data collection. The audit reported in Section 3.10 shows that a small proportion of the test images have a byte identical or a perceptually near-identical counterpart on the training side. The analytic bound derived in that section shows that such redundancy can account for at most a few hundredths of a percentage point of the reported accuracy, so the conclusions of this study are not affected. The presence of near-duplicates is nevertheless a property of the source collection that any study based on it inherits, and future work would benefit from a collection in which every image is guaranteed to originate from a distinct leaf.

The unseen crop evaluation was conducted on maize and potato images that, like the training images, were acquired under controlled laboratory conditions with uniform illumination and a simple background. The acquisition condition statistics reported in **Table 7** confirm that the two collections are similar in this respect. The transfer reported in Section 3.6 and Section 3.11 should therefore be read as transfer between datasets acquired under matched conditions, and not as evidence of robustness under field conditions. Matched acquisition conditions are characteristic of the public cross-crop collections used in this line of work (Paymode and Malode, 2022). The perturbation probe of **Table 8** shows that accuracy decreases systematically as the images are moved away from those conditions. Establishing field robustness requires images collected in commercial crops with natural illumination, occlusion, mixed infections and variable phenological stages, and this remains the most important next step for this line of work.

### 4.10 Computational cost and code availability

The computational cost of the proposed model was measured on a single workstation: an NVIDIA GeForce RTX 3060 with 12 GB of memory, CUDA 12.6, PyTorch 2.14.0 and torchvision 0.29.0, with a 300 by 300 pixel input and a batch size of one. Under these conditions the proposed model has 15.64 million parameters, 1.17 GMACs (2.33 GFLOPs), a size of 59.7 MB, and an inference latency of 20.27 ms. At 15.64 million parameters and 1.17 GMACs it carries about 2.9 times the parameters and about 5.3 times the arithmetic of MobileNetV3-Large, and about 60% of the parameters and about 30% of the arithmetic of ResNet-50, so it is a model of moderate computational cost. **Table 11** reports these measurements alongside four reference architectures.

**Table 11:** Parameters, MACs, FLOPs, size and latency (proposed and four references).

Model	Parameters (M)	MACs (G)	FLOPs (G)	Size (MB)	Inference (ms)
ResNet-50	25.56	3.86	7.71	97.5	8.39
MobileNetV3-Large	5.48	0.22	0.44	20.9	8.53
EfficientNet-B0	5.29	0.39	0.78	20.2	10.94
EfficientNet-B3 backbone	12.23	0.97	1.93	46.7	17.36
Proposed model	15.64	1.17	2.33	59.7	20.27

About code availability, the implementation, including the training and evaluation scripts, the linear-probe transfer script, the perturbation evaluation script, the group definitions and the five partition index lists, and the configurations of the four ablation variants, is available at https://github.com/czpbest/ccldr. The trained weights are archived at https://github.com/czpbest/ccldr/releases/tag/v1.0.

## 5 Conclusions

This study tests the functional-domain separation hypothesis through systematic architecture design and experimentation, providing computational evidence for cross-family conservation of plant pathological features as a contribution to understanding plant-biotic interactions. The sub-additive interaction between channel calibration and spatial interaction domains (the measured combined gain falls 0.04 percentage points below the additive prediction of the individual gains) provides supporting evidence for implicit feature disentanglement (Bengio et al., 2013), without confirming that the two functional domains operate on non-overlapping factors of variation. Combined with the observation that cross-crop confusion accounts for only one of the 5,586 test images in the confusion matrix, this offers supporting evidence rather than a causal chain from architectural design principle through representation disentanglement to classification performance. The cross-family generalization from Solanaceae, Vitaceae, and Rosaceae to Poaceae (maize), with potato remaining within Solanaceae, with an accuracy of 88.99% on maize and 95.23% on potato under matched acquisition conditions, provides computational evidence for cross-family conservation of visual pathological features, but is reported as a descriptive finding rather than as a mechanistic confirmation of conserved pathogenic pathways. Across the five independent group-aware partitions the architecture attains a mean accuracy of 99.93% with 19 misclassified test images among 27,937, which indicates the practical value of functional-domain separation. Beyond architectural design, this computational evidence contributes to the broader understanding of plant-biotic interactions by reporting the cross-family conservation of pathological signatures observed in the data, without independently validating the molecular plant pathology prediction. These figures rest on partitions in which images derived from the same source leaf are kept together, and a duplicate and near-duplicate audit places the inflation attributable to data redundancy at a few hundredths of a percentage point, so the in-domain value is not a product of partitioning an augmented corpus.

The principal contribution of this study is to show that a theoretically grounded architectural design is consistent with, rather than causal of, cross-species generalization through representation disentanglement, providing computational support for cross-family conservation of plant pathological features. This work demonstrates that theoretically grounded architectural design, rather than empirical component accumulation, can yield architectures that capture transferable phytopathological signatures across plant families. The sub-additive interaction between functional domains offers a measurable criterion for examining whether architectural designs achieve the intended separation of disease-specific and crop-specific factors, subject to the between-partition variability reported in Section 3.5. These findings open a new direction for computational plant pathology, where architectural design principles are derived from theoretical foundations rather than empirical observations, potentially accelerating the development of cross-species transferable recognition systems for plant disease surveillance.

These findings provide empirical support for the hypothesis that functional-domain separation induces implicit feature disentanglement, enabling architectures to capture transferable phytopathological signatures shared across the tested plant families. Future work may extend the framework to further plant families and to bacterial and viral pathogens, test whether the complementarity principle generalizes to alternative recalibration or token-mixing mechanisms (Hu et al., 2020; Tolstikhin et al., 2021), quantify disentanglement directly with mutual information estimation or probe classifiers, and repeat the ablation over multiple seeds to test the significance of the sub-additive interaction. Practical extensions include the few-shot (Rezaei et al., 2024), federated (Aggarwal et al., 2024), hyperspectral (Li et al., 2023, 2024) and incremental-learning (Mu et al., 2024) schemes. Biological validation through controlled cross-family inoculation, combined with molecular analysis of conserved defense pathways, would provide independent confirmation.

Beyond its scientific reading, the frozen-backbone protocol has a practical consequence: when a new crop is introduced, only the linear head is retrained, so cross-crop adaptation is cheap relative to training a network from scratch and can be extended to further crops with limited labelled data. This advantage is confined to the frozen regime: on the single partition where full fine-tuning was tested, the proposed representation and an ImageNet-initialized control differ by less than the seed-to-seed spread, so no general advantage over that control is claimed.

## Data Availability

The original contributions presented in the study are included in the article/supplementary material. Further inquiries can be directed to the corresponding author.
